# State and parameter estimation for a neural model of local field potentials

**DOI:** 10.1007/s00285-026-02451-w

**Published:** 2026-10-03

**Authors:** Daniele Avitabile, Gabriel J. Lord, Khadija Meddouni

**Affiliations:** 1 Amsterdam Center for Dynamics and Computation, VU Amsterdam, Amsterdam, The Netherlands; 2 Inria Math Neuro Team, Inria Branch at the University of Montpellier, Montpellier, France; 3 Department of Mathematics, IMAPP, Radboud University, Nijmegen, The Netherlands

**Keywords:** Wilson–Cowan–Amari model, Bayesian inference, Particle filters, Local field potential, Parameter estimation, 62F15, 65C05, 93E11, 62M20

## Abstract

The study of cortical dynamics during different states such as decision making, sleep and movement, is an important topic in Neuroscience. Modelling efforts aim to relate the neural rhythms present in cortical recordings to the underlying dynamics responsible for their emergence. We present an effort to capture certain features of the neural activity from the cortex of a mouse during natural sleep, obtained through local field potential measurements. Our approach relies on using a discretized Wilson–Cowan Amari neural field model for neural activity, along with a data assimilation method that allows the Bayesian joint estimation of the state and parameters. We demonstrate the feasibility of our approach on synthetic measurements before applying it to a dataset available in literature. Our findings suggest the potential of our approach to characterize the parameters of the external forcing in the neural activity model, while simultaneously inferring a state that aligns with the observed signal.

## Introduction

Data assimilation is a form of inverse problem that enhances model prediction using partial and noisy observations, and in which the unknown quantity is a dynamical system’s state, its control parameters, or a combination of these quantities. This approach to model forecast, parameter inference, and uncertainty quantification has been the subject of an intense work over the past decade, ranging from theoretical foundations to domain-specific studies in engineering, physics, and biology (Smith 2014; Law et al. 2015; Nakamura and Potthast 2015; Reich and Cotter 2015; Sanz-Alonso et al. 2023).

The present paper tests and showcases the data assimilation methodology in the context of brain dynamics. Our long-term motivation is to develop a numerical tool to gain a better understanding of how cortical and limbic brain regions interact during sleep, when memories are thought to consolidate (Sanda et al. 2020). This is a compelling application for data assimilation, because experimental observations of brain activity are available for the cortex (Pedrosa et al. 2022), and one is interested in describing the coupling with hidden limbic regions. Thus it is interesting to model such interactions, even in a simple form, and test them against the partial observations of the system, in the upper (cortical) layers.

Deterministic inverse problems in which the unknown quantity is a resting state or a static parameter typically suffer from ill-posedness (Curtis 2002; Kirsch 2011), which can be resolved by appealing to regularization techniques (see for example (Willoughby 1979; Kirsch 2011)) or to a Bayesian formulation as described in Joel and Franklin (1970); Stuart (2010); Kaipio and Somersalo (2005); Dashti and Stuart (2017).

Within the data assimilation framework, in which data is time-dependent, we distinguish between two categories: methods such as 4D-Var where the state is estimated over a whole time window of observations, and sequential methods such as Kalman or particle filters which incorporate the observations sequentially. The latter is the approach we follow in this paper.

Our aim is to examine Bayesian joint estimation of parameters and prediction of state from the local field potential measurements. Our approach relies on the use of the nested particle filter from Crisan and Míguez (2013); Miguez et al. (2023) on a nonlinear state-space system originating from the discretized deterministic Wilson–Cowan–Amari neural field originally proposed in Wilson and Cowan (1973); Shun-Ichi Amari (1977). This equation models heuristically spatially-dependent brain activity in a cortical domain using coarse activity variables, their nonlocal synaptic interaction, and the presence of an external forcing, which may come from other (cortical or non-cortical) regions, from an external stimulation, or compensate for missing biological terms. We henceforth refer to any of these sources as *external forcing*, and we stress that the model employed here is not sufficiently granular to distinguish between them. The present paper focuses on the numerical aspects of the inversion process, and our model choice has been dictated by mathematical structure and wide applicability, rather than biological accuracy.

Although the dynamics of this model have been studied extensively (Ermentrout and Cowan 1979; Paul and Bressloff 2011; Coombes 2005; Kilpatrick and Bressloff 2010), there are far fewer works that tackle the inverse problem of estimating state or model parameters (Steven et al. 2007; Roland Potthast and Peter beim Graben 2009; Alswaihli et al. 2018). In the context of joint parameter and state estimation, related works include Kulikova and Kulikov (2023), in which the authors derived a continuous-discrete extended Kalman filter for simultaneous state and parameter estimation for stochastic dynamic neural field models and demonstrated the robustness of their approach on synthetic measurements. The authors of Steven et al. (2007) used the unscented Kalman filter to estimate both state and parameters of a spatiotemporal excitable cortical model and showed its ability to track spiral waves. In a subsequent work, Sauer and Schiff (2009) introduced the consensus set method with an ensemble Kalman filter applied to a homogeneous Wilson-Cowan model to effectively track the state and infer parameters in both synthetic and experimental measurements. Recently, state and parameter estimation for the model has been studied from a theoretical viewpoint and with synthetic data in Pieper-Sethmacher et al. (2025), in which data assimilation is introduced with a form of nudging, called guided process.

The present paper differs from existing literature as it uses experimental observations with a nonlinear filter, which does not resort to linearised dynamics. Further, we test the performance of the spatio-temporal reconstruction against the dataset, and use synthetic data to show how the methodology accounts for epistemic uncertainty (model imperfections). While this paper is concerned mostly with the numerics of data assimilation, our results show the potential of this framework to quantify the influence of external forcing on the modelled cortical activity, while matching faithfully its experimental observations.

The paper is organized as follows. In Sect. 2 we present the setting for the estimation problem of interest including a description of the cortical model and a background on Bayesian estimation. Section 3 provides the results of this approach on synthetic data. We present the experimental cortical data in Sect. 4 and the estimation results in Sect. 5. We conclude in Sect. 6 with a summary of this work, and we detail some additional computational experiments in the Appendices.

## Problem setting and background

### The neural field model

We study the data assimilation problem on a discrete version of a one-population Amari neural field model

1 $$\begin{aligned} \tau \frac{\partial u(r,t)}{\partial t}=-u(r,t)+\int _D w(r,s)\sigma (u(s,t))ds +\Xi (r,t;\theta ,a), \qquad r \in D, \qquad t \ge 0 \end{aligned}$$

with initial condition $u(r,0)=u_0(r)$, posed on a one-dimensional, bounded cortical domain $D \subset \mathbb {R}$. The model describes the evolution of the population’s neural activity *u* at cortical position *r* and time *t*. The population has nonlinear firing rate σ, and typical response time τ, while *w*(*r*, *s*) models synaptic connections from point *s* to point *r* in the cortex. Further, the population is subject to a spatio-temporal external forcing Ξ, which depends upon a set of control parameters $\theta \in \mathbb {R}^{d_\theta }$ that we wish to infer. The forcing may also possibly depend on other parameters *a* that we keep fixed. We note that the functions *w* and σ also depend on the parameters, but we omit this dependence for the sake of conciseness.

We consider synaptic connections described by a Mexican hat kernel, which can model short-range excitation and long-range inhibition,

2 $$\begin{aligned} w(r,s)=A_{ex}\exp \left( {\frac{-\Vert r-s \Vert ^2}{\ell _{ex}^2}}\right) -A_{in}\exp \left( {\frac{-\Vert r-s \Vert ^2}{\ell _{in}^2}}\right) , \end{aligned}$$

and a sigmoidal firing rate function with threshold μ and slope λ

3 $$\begin{aligned} \sigma (u)=\frac{1}{1+\exp (-\lambda (u-\mu ))}. \end{aligned}$$

In this paper, our aim is to characterize the external forcing Ξ by employing a sequential data assimilation method. To this end, we fix τ and all the parameters appearing in *w* and σ to the values given in Table 1; we are interested in inferring parameters for the forcing Ξ and simultaneously estimating a discrete approximation of the state variable u(·,t) from noisy observations.

Strictly speaking, the model we use is a spatio-temporal discretisation of the continuum model (1), for specific choices of the spatio-temporal stimulus Ξ which are described later, in Sects. 3 and 5, as they change depending on the setup of the problem and numerical experiments. We highlight, however, that we do not quantify how spatial and temporal discretizations affect the data assimilation process, but we rather take the discrete model as the starting point for our investigation. The rest of this section is devoted to describing this discrete model.

We take D=[0,L] and discretize space using *J* evenly spaced nodes $r_0,\ldots , r_{J-1} \in D$, with $r_j = j \Delta r$ for j=0,…,J-1. Further, we approximate the integral using the standard composite trapezium rule for the quadrature with nodes $\{r_j\}$ and weights $\{ b_j \}$ given by $b_0 = b_{J-1} = {\Delta r}/2$, and $b_j = {\Delta r}$ otherwise. Let $U(t)\in \mathbb {R}^J$ be the vector approximating $\big (u(r_0,t),\ldots ,u(r_{J-1},t)\big )^T$, $W \in \mathbb {R}^{J \times J}$ the matrix with components $W_{ij}=w(r_i,r_j)b_j$, $S:\mathbb {R}^J\mapsto \mathbb {R}^J$ is the multidimensional analogous of the firing rate σ, *S*(*U*(*t*)) and I(t;θ,a) are the vectors in $\mathbb {R}^J$, such that *S*(*U*(*t*)) approximates $\big (\sigma (u(r_0,t)),\ldots ,\sigma (u(r_{J-1}))\big )^T$ and $I(t;\theta ,a)=\big (\Xi (r_0,t;\theta ,a),\ldots ,\Xi (r_{J-1},t;\theta ,a)\big )^T$. This leads to the semi-discrete model

4 $$\begin{aligned} \tau \frac{dU(t)}{dt}=-U(t)+WS(U(t))+I(t; \theta ,a), \qquad U(0) = U_0 \in \mathbb {R}^J, \end{aligned}$$

**Table 1:** Model parameters used throughout this paper unless stated otherwise

Parameter	Description	Value(unit)
τ	membrane time constant of neurons	$0.03~(\textrm{s})$
$\ell _{ex}$	spatial spread of of excitatory connections (short-range)	$0.5~(\textrm{mm})$
$\ell _{in}$	spatial spread of of inhibitory connections (long-range)	$1~(\textrm{mm})$
$A_{ex}$	excitatory connections strength	$10~(\textrm{mV})$
$A_{in}$	inhibitory connections strength	$6~(\textrm{mV})$
λ	steepness of the sigmoidal, controls how sensitive the firing rate is	$5~(\textrm{mV})^{-1}$
μ	activity threshold for neural firing	$0.5~(\textrm{mV})$

As common in sequential data assimilation, we work with a discrete-time approximation to the dynamical system of Eq. 4. Here we integrate Eq. 4 in time using a standard explicit Euler method on a uniform grid of the temporal domain [0, *T*], hence for a given $K\in \mathbb {N}$ we let $t_k=k{\Delta t}$ with k=0,1,…,K and ${\Delta t}=T/K$, and consider the approximating sequence $U_k \approx U(t_k)$ given by

5 $$\begin{aligned} U_{k+1}=\Psi _k(U_k, \theta ) := U_k+\frac{\Delta t}{\tau }\big (-U_k+WS(U_k)+I(t_k; \theta ,a)\big ), \quad U_0 = U(0), \end{aligned}$$

which we take as our model of cortical dynamics. Note that $\Psi _k$ also depends on other non-inferred parameters such as *a* and the parameters appearing in the functions *w* and σ. For clarity of notation, we omit this dependence when no ambiguity arises.

### Background on particle filters and Bayesian estimation

#### The data assimilation framework

Data assimilation combines uncertain estimations from a model with noisy (and sometimes partial) observations to provide an estimate of an unobserved (hidden) dynamical state. Consider two stochastic processes $\{X_k\}_{k=0}^{{\infty }} \subset \mathbb {R}^{d_x}$ and $\{Y_k\}_{k=1}^{{\infty }} \subset \mathbb {R}^{d_y}$, with realisations $x_k$, $y_k$, which describe the hidden state and measurements, respectively. We adopt the standard notation $x_{k:l}=\left\{ x_k,x_{k+1},\ldots ,x_{l}\right\}$ and similarly for $y_{k:l}$. At time step k≥0, $X_k \in \mathbb {R}^{d_x}$ represents the hidden state to be estimated and for k≥1, $Y_k \in \mathbb {R}^{d_y}$ represents the measurements. They are assumed to satisfy

6 $$\begin{aligned} X_{k}&=F_{k}(X_{k-1},\theta ,\eta _{k}),&k \ge 1 \end{aligned}$$

7 $$\begin{aligned} Y_{k}&=G_{k}(X_k,\gamma _k),&k \ge 1. \end{aligned}$$

The *state Eq. * (6) describes how the hidden state $X_k$ evolves over time and the *observation Eq. * (7) links the hidden state to the measurement $Y_k$. The model function $F_k:\mathbb {R}^{d_x} \times \mathbb {R}^{d_{\theta }} \times \mathbb {R}^{d_x}\rightarrow \mathbb {R}^{d_x}$ and observation function $G_k:\mathbb {R}^{d_x}\times \mathbb {R}^{d_y}\rightarrow \mathbb {R}^{d_y}$ are assumed to be known. The process noise, $\eta _k \in \mathbb {R}^{d_x}$, represents the model uncertainty and is assumed to have a known probability distribution. Similarly $\gamma _k\in \mathbb {R}^{d_y}$ is the observation noise, also with a known distribution. We assume that for k≠l, the process (observation) noise vectors $\eta _k$, $\eta _{l}$ ($\gamma _k$, $\gamma _{l}$) are mutually independent and are mutually independent of the initial state $X_0$. Further, for all *k*, *l*, $\eta _k$ and $\gamma _{l}$ are mutually independent. Equations (6) and (7) form a *state-space* system. While the function $F_k$ depends on the control parameters θ, we may omit this dependence wherever possible from now on.

In this paper, we assume additive noise in the state and observation equations, and use a linear observation operator for the state variables of Eq. (5), that is, we consider

8 $$\begin{aligned} \begin{aligned} X_k&=\Psi _{k-1}(X_{k-1},\theta )+Q\eta _{k} \\ Y_k&=G X_k+R\gamma _k, \end{aligned} \end{aligned}$$

for k=1,…,K, where $d_x=d_y=J$, $G\in \mathbb {R}^{J\times J}$ is an observation matrix that is specified later for each experiment, $\gamma _k,\eta _{k}\sim \mathcal {N}(0,{{\,\textrm{Id}\,}}_J)$ are independent and identically distributed normal random variables with values in $\mathbb {R}^J$, and $Q,R \in \mathbb {R}^{+}$ are the noise intensities.

With a slight abuse of notation we use π(x) and π(y) to denote the probability densities of *X* and *Y* respectively, even though they are different functions (the argument is used to distinguish them) and assume that the probability distributions of $X_k$ and $Y_k$ are absolutely continuous with respect to the Lebesgue measure ($\mu _{X_k}(dx_k)=\pi (x_k) dx_k$ and similarly for $Y_k$).

It is standard to assume $\{X_k\}_{k=0}^{{\infty }}$ is a Markovian process

9 $$\begin{aligned} \pi (x_{k+1}|x_{0:k})=\pi (x_{k+1}|x_k), \end{aligned}$$

and depends on the past observations $y_{1:k}$ only through its own history

10 $$\begin{aligned} \pi (x_{k+1}|y_{1:k})=\pi (x_{k+1}|x_k). \end{aligned}$$

Similarly $\{Y_k\}_{k=1}^{{\infty }}$ is assumed to be a Markovian process with respect to the history of $\{X_k\}_{k=0}^{{\infty }}$, such that

11 $$\begin{aligned} \pi (y_{k}|x_{1:k})=\pi (y_{k}|x_k). \end{aligned}$$

#### Bayesian filtering

In the Bayesian setting, the sequential filtering problem is formulated as determining $\pi (x_k|y_{1:k})$ the posterior probability density of the state $X_k$ at time step *k*. Bayesian filtering methods operate sequentially by incorporating each new observation using Bayes’ formula. At timestep k+1, the posterior density of the state from the previous timestep $\pi (x_k|y_{1:k})$ is evolved in time through the state Eq. (6), leading to the prediction density $\pi (x_{k+1}|y_{1:k})$, so

12 $$\begin{aligned} \pi (x_{k+1}|y_{1:k})=\int \pi (x_{k+1}|x_k)\pi (x_{k}|y_{1:k})dx_k. \end{aligned}$$

This prediction is then taken as a prior in the Bayes’ formula and is updated or corrected with the new observation $y_{k+1}$ using the likelihood $\pi (y_{k+1}|x_{k+1})$ derived from the observation Eq. (7), that is

13 $$\begin{aligned} \pi (x_{k+1}|y_{1:k+1}) =\frac{\pi (y_{k+1}|x_{k+1})\pi (x_{k+1}|y_{1:k})}{\pi (y_{k+1}|y_{1:k})}, \end{aligned}$$

where

$$\begin{aligned} \pi (y_{k+1}|y_{1:k})=\int \pi (y_{k+1}|x_{k+1})\pi (x_{k+1}|y_{1:k})dx_{k+1}. \end{aligned}$$

When the state-space model is linear and Gaussian, the densities in (12) and (13) can be computed exactly leading to the Kalman filter (Kalman 1960). In the presence of non-linearities, several approximations exist such as the extended Kalman filter (Jazwinski 1970; Gelb 1974) or the unscented Kalman filter (Wan and Van Der Merwe 2000). Particle filters (Neil 1993; Doucet et al. 2001) are an alternative approach and are a class of filters that do not require any additional assumptions of linearity or Gaussianity of the state-space model. They rely only on Monte Carlo methods and importance sampling to approximate the posterior density through an ensemble of random samples, referred to as particles. In practice, particle filters implement the Bayesian filtering Eqs.  (12) and (13): they rely on having an ensemble of *M* particles $\{x_k^1,\cdots ,x_k^M\}$ (positions in the state space at time *k*) along with their weights $\{\omega _k^1,\cdots ,\omega _k^M\}$ (the probability that a particle represents the true position) as an approximation to the density $\pi (x_k|y_{1:k})$. The aim is to first produce an ensemble of particles to approximate the prediction measure $\pi (x_{k+1}|y_{1:k})$ and then another ensemble for the posterior $\pi (x_{k+1}|y_{1:k+1})$ with updated weights that reflect how the particles match the observations. The first step uses the state Eq. (6) to approximate Eq. (12), while the second makes use of the prior distribution as an importance sampling distribution in order to make the update formula of the weights in Eq. (13) sequential. A direct implementation will run into a problem of degeneracy: as time progresses, only few particles will have significant weight while the majority of the particles end up with extremely low weights. This implies that only a few particles contribute significantly to the approximation of the posterior distribution, effectively reducing the ensemble diversity and the quality of the estimation (since not all regions of the posterior are sampled). In fact, at time step *k* the posterior distribution is approximated through a weighted empirical measure

$$\begin{aligned} \pi (x | y_{1:k})\approx \sum _{m=1}^{M} \omega _k^m \delta (x -x_k^m), \end{aligned}$$

where δ is the Dirac delta. From which we have the estimate $\mathbb {E}[g(x)|y_{1:k}]\approx \sum _{m=1}^{M} \omega _k^m g(x_k^m)$, for any integrable function of interest *g* (e.g. the posterior mean or variance). The quality of such estimates relies on the particles covering the relevant regions of the posterior and on their weights corresponding to their likelihood. This fails when the ensemble is composed of few particles with non-negligible weights. Indeed, in the worst case, the ensemble may collapse to a single particle with non-negligible weight. To counter this, a resampling step is normally added, so that particles that do not match the observation (and thus have extremely low weight) are thrown away and replaced with duplicates of the particles with higher probability. In practice, we resample our ensemble of particles each time the effective number of particles $M_{\text {eff}}$, approximated by $M_{\text {eff}}:=1/\sum _{m=1}^{M} (\omega _k^{m})^2$, falls below a threshold of *M*/2. $M_{\text {eff}}$ can be interpreted as the number of independent, equally-weighted samples that would give the same quality approximation. A high $M_{\text {eff}}$ indicates a diverse particle ensemble, for example, when all the particles are uniformly weighted with 1/*M*, $M_{\text {eff}}=M$. On the other hand, when all weights are zero except for a single particle with weight one, $M_{\text {eff}}=1$ indicating that the entire posterior is represented by a single particle, regardless of how many particles were nominally propagated. The resampling step consists in drawing the particles with replacement according to their normalized weight. Based on the findings of Douc and Cappe (2005); Kuptametee and Aunsri (2022), we use stratified resampling in our numerical simulation as given in Kitagawa (1996).

Algorithm 1 presents a straightforward implementation of this instance of particle filters, known as the sequential importance resampling (SIR) particle filter.

**Algorithm 1:**
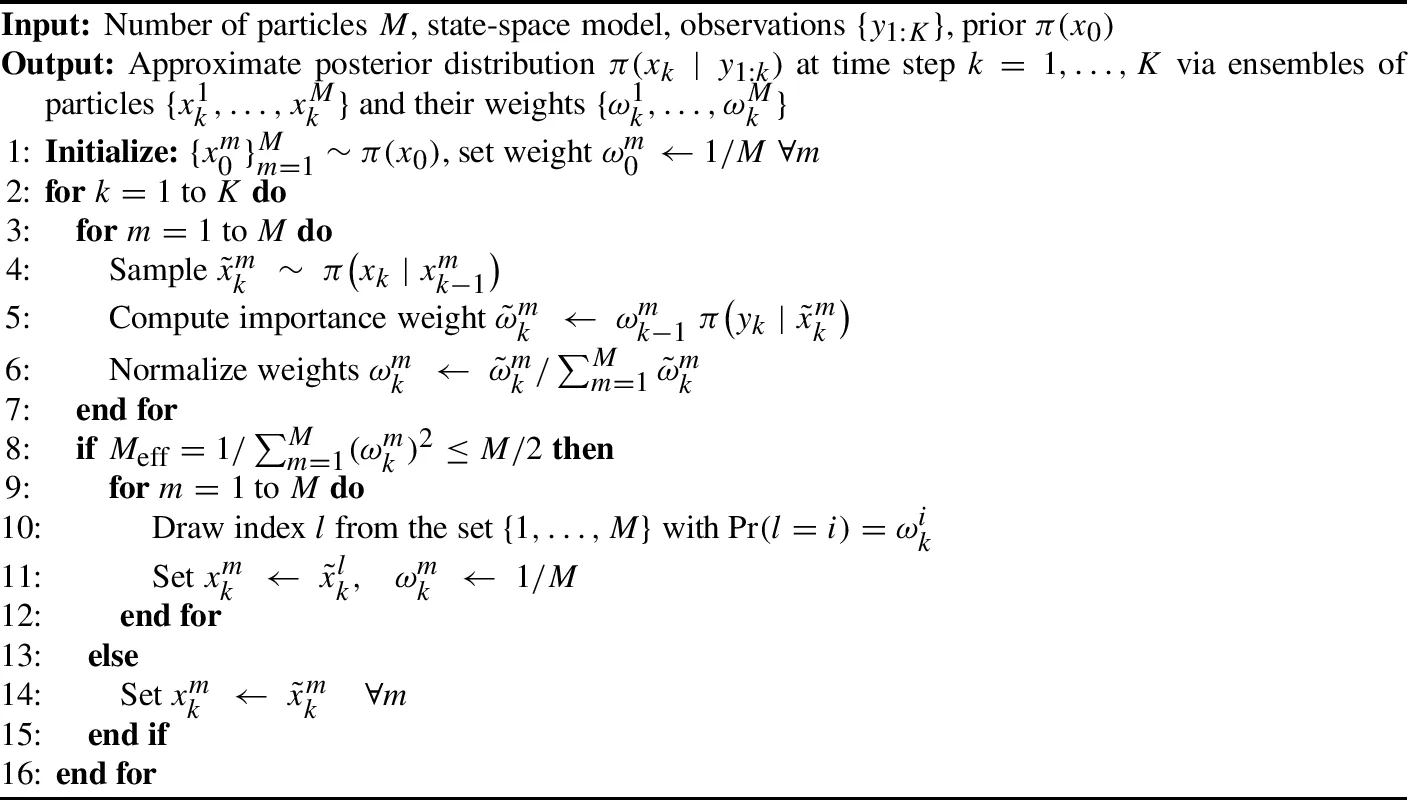
SIR

#### Nested particle filters

In the previous sections, it was implicitly assumed that the model function $F_k$ is fully known and depends only on the state itself. However, in most applications, the exact values of the model parameters θ are not known and need to be estimated along with the state.

One approach to perform a joint state and parameter estimation is the nested particle filter presented in Crisan and Míguez (2013); Miguez et al. (2023), which we adopt here. This nested filter is a recursive algorithm that can be described as two layers of particle filters. In the outer layer, *N* particles for the parameters are generated, and for each particle, an inner filter from Algorithm 1 is used to estimate the state with *M* particles for each of the *N* parameter particles. For the sake of completeness, we present the method in Algorithm 2.

The jittering or rejuvenation step of the algorithm consists in drawing new parameter samples from a kernel to avoid the collapse of the ensemble of particles for the parameters. The new sample $\overline{\theta }_k^i$ must be close enough to the previous sample $\theta _{k-1}^i$, so that the filter approximation of $x_{k-1}$ computed for $\theta _{k-1}^i$ can be used as a particle approximation of the filter for the new sample $\overline{\theta }_k^i$. Following Crisan and Míguez (2013); Miguez et al. (2023), we use the following kernel

14 $$\begin{aligned} \kappa _k^N(\theta )=(1-\epsilon _N)\delta _{\theta }+\epsilon _N\mathcal{T}\mathcal{N}(\theta ,{\sigma ^2}_{\theta }^N, A_{\theta }, B_{\theta }), \end{aligned}$$

where $\epsilon _N=\frac{1}{\sqrt{N}}$ is the probability of jittering a particle, $\mathcal{T}\mathcal{N}$ is the truncated normal distribution with mean θ, variance ${\sigma ^2}_{\theta }^N \propto N^{-3/2}$ and a support $[A_{\theta }, B_{\theta }]$ that varies for each component of the parameter vector θ.

We consider the empirical posterior means

$$\begin{aligned} \mathbb {E}[\theta _k|y_k]\approx \frac{1}{N}\sum _{n=1}^N \theta _k^n, \qquad \mathbb {E}[x_k|y_k]\approx \frac{1}{NM}\sum _{n=1}^N\sum _{m=1}^M x_k^{(n,m)} \end{aligned}$$

as our estimates at time step *k* of the parameters and state respectively.

**Algorithm 2:**
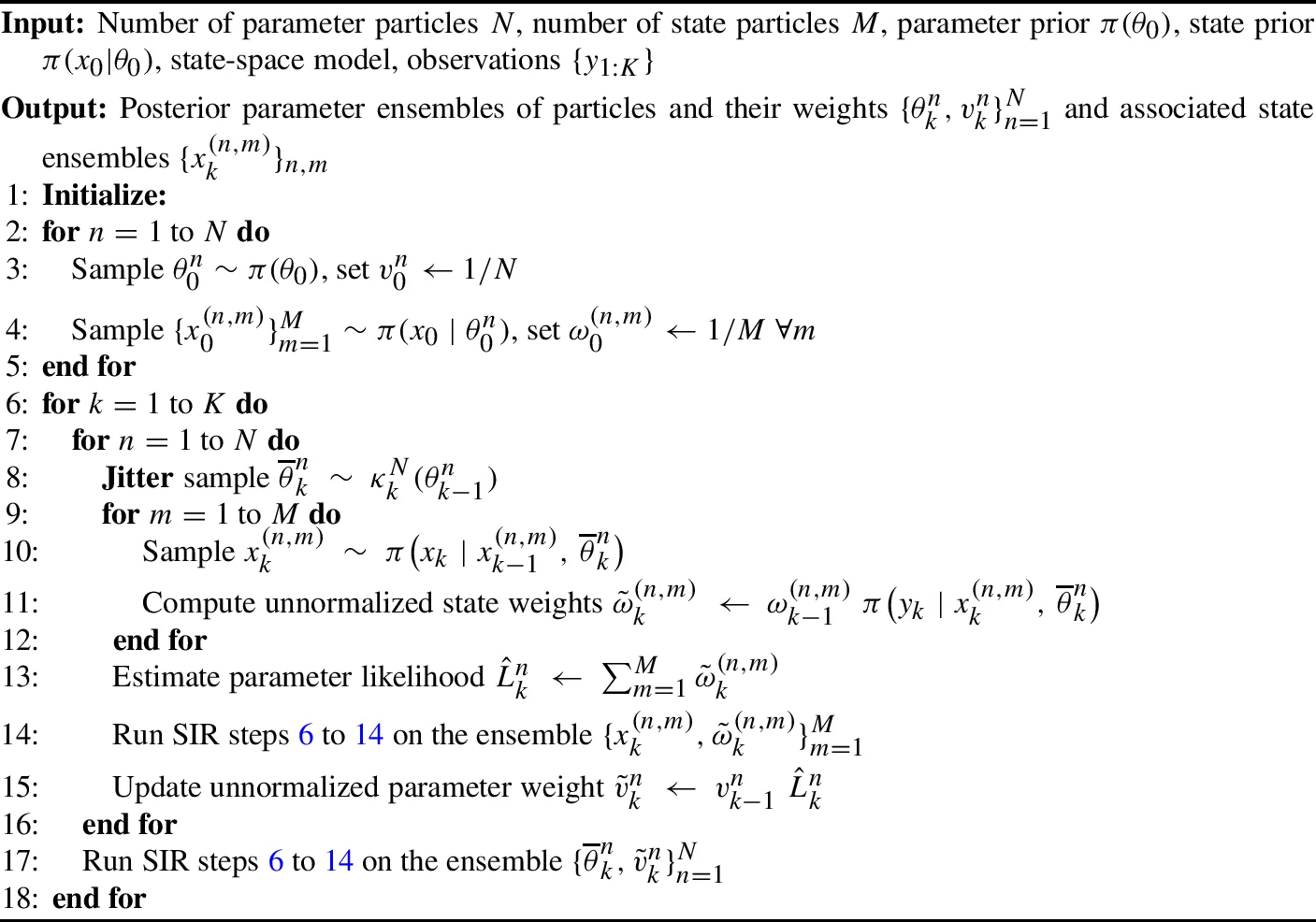
Nested Particle Filter

## Nested particle filter applied to synthetic data

Before using the particle filter on cortical data, we test the data-assimilation algorithm on synthetic data. We consider the states $\{ U^*_k \}_{k = 0}^{K^*} \subset \mathbb {R}^{J^*}$ generated by model (5), in which the mapping $\Psi ^*_k(U,\theta ^*)$ is specified via a matrix $W^* \in \mathbb {R}^{J^* \times J^*}$ and input $I^*(t; \theta ^*, a^*) \in \mathbb {R}^{J^*}$.

The input features 3 inferable parameters $\theta ^* = (A^*, \nu ^*, f^*)$ as well as an additional parameter $a^*$, and has components $\left( \Xi (r_j,t,\theta ^*,a^*)\right) _{j=0}^{J^*-1}$ at time *t* and spatial nodes $r_j=j{\Delta r}$ where

$$ \Xi (r,t,\theta ^*,a^*) = A^* \cos 2 \pi \bigl (\nu ^* r - (f^* + a^* t) t\bigr ). $$

The external input models a *chirp travelling wave* with amplitude $A^*$ (mV), spatial frequency $\nu ^*$ ($\text {mm}^{-1}$), and time-dependent frequency $f(t) = (f^* + a^*t)$, hence $f^*$ (Hz) is the initial temporal frequency and $a^*$ (Hz/s) is the chirp rate.

The data-assimilation task aims to reconstruct $\{ U^*_k \}$ and infer $\theta ^*$ under noisy observations, as well as (epistemic) model uncertainty. To this end, we setup system (8) as follows:

- *Noisy observations.* The observations $\{ Y_k \}_{k=1}^K \subset \mathbb {R}^J$ are obtained by sampling the original signal $\{ U^*_k \}_{k=0}^{K^*} \subset \mathbb {R}^{J^*}$ on a coarse spatio-temporal grid ($K < K^*$, $J < J^*$), and by adding noise.
- *Model uncertainty.* When performing data assimilation, one has typically only limited knowledge of the model that generated the data. To mimic this scenario, we use a mapping $\Psi _k(\cdot , \theta )$ in the state space system (8) that differs from $\Psi _k^*(\cdot ,\theta ^*)$ used to generate the original state in two ways: firstly, the matrix $W\in \mathbb {R}^{J \times J}$ and input vector $I(t;\theta ,a) \in \mathbb {R}^J$ sample the functions *w* and Ξ on the coarse spatial grid; secondly, the input vector I(t;θ,a) has components $\bigl (\Xi (r_j,t,\theta ,0)\bigr )_{j=0}^{J-1}$, that is, the model used for inference has null chirp rate and constant frequency *f*, while the one giving rise to the observations has a time-dependent frequency. Although it is beyond the scope of this work and not necessary for the comprehension and interpretation of the results presented here, we provide in Appendix A a minimal quantification of the relative influence of the inferable parameters θ through first and total order Sobol indices.

We present numerical evidence that the nested particle filter algorithm generates estimating sequences of states $\{ X_k \}$ and empirical posterior means of parameters $\{ \theta _k \}$ such that: the state $\{ X_k\}$ is close to $\{ U^*_k \}$ on the coarse spatio-temporal grid; the sequence $\{ (A_k,\nu _k) \}$ converges to $(A^*,\nu ^*)$; the sequence $\{ f_k \}$ does not converge to a limit, but nevertheless provides some information on the original model. Our numerical experiment is setup as follows.

- *Generating the signal.* To generate $\{ U^*_k \}$ we simulate (5) on $(r,t) \in [0,L] \times [0,T]$ with $L = 10\text {mm}$ and T=100s, using a grid with $J^* = 500$ spatial points, and $K^* = 20000$ time points. The initial condition is given by $u_0(r) = \sin (\pi r)$, and model parameters are fixed as
  $$ (\theta ^*,a^*) = (A^*,\nu ^*,f^*,a^*)=(1,0.1,0.5,-0.005). $$
- *Generating observations.* We interpolate $\{ U^*_k \}$ on a coarse grid of [0,L]×[0,T] with J=30 and K=10000, and obtain an intermediate sequence $\{ \bar{U}_k \}_{k =0}^K \subset \mathbb {R}^{J}$. The observation vectors satisfy
  $$ Y_k = \bar{U}_{k} + \varepsilon \xi _k, \qquad \xi _k {\mathop {\sim }\limits ^{i.i.d}} \mathcal {N}(0,{{\,\textrm{Id}\,}}_J), \qquad k = 1,\ldots ,K, \qquad \varepsilon = 0.5. $$
- *Running the nested particle filter.* In the state-space system (8) we set
  $$ Q = 0.1, \qquad R = 0.5, \qquad \eta _k, \gamma _k {\mathop {\sim }\limits ^{i.i.d}} \mathcal {N}(0,{{\,\textrm{Id}\,}}_J), \qquad k = 1,\ldots ,K. $$
  Further, we run Algorithm 2 once using N=M=500 particles with uniform priors on the inferable parameters given by:
  $$\begin{aligned} \theta = (A,\nu ,f)\sim \mathcal {U}([0,10])\otimes \mathcal {U}([0,1])\otimes \mathcal {U}([0,1]). \end{aligned}$$
  Priors on the state *X* are samples from a discretized mean-zero Gaussian process with a squared exponential covariance $\mathcal{G}\mathcal{P}\big (0,k(r,s)\big ),$ where $k(r,s)=2\exp {\left( -(r-s)^2/2\right) }$, hence we set
  15 $$\begin{aligned} X_0\sim \mathcal {N}(0,C), \qquad C\in \mathbb {R}^{J\times J}, \qquad C_{i,j}=k(r_i,r_j). \end{aligned}$$

**Fig. 1:**
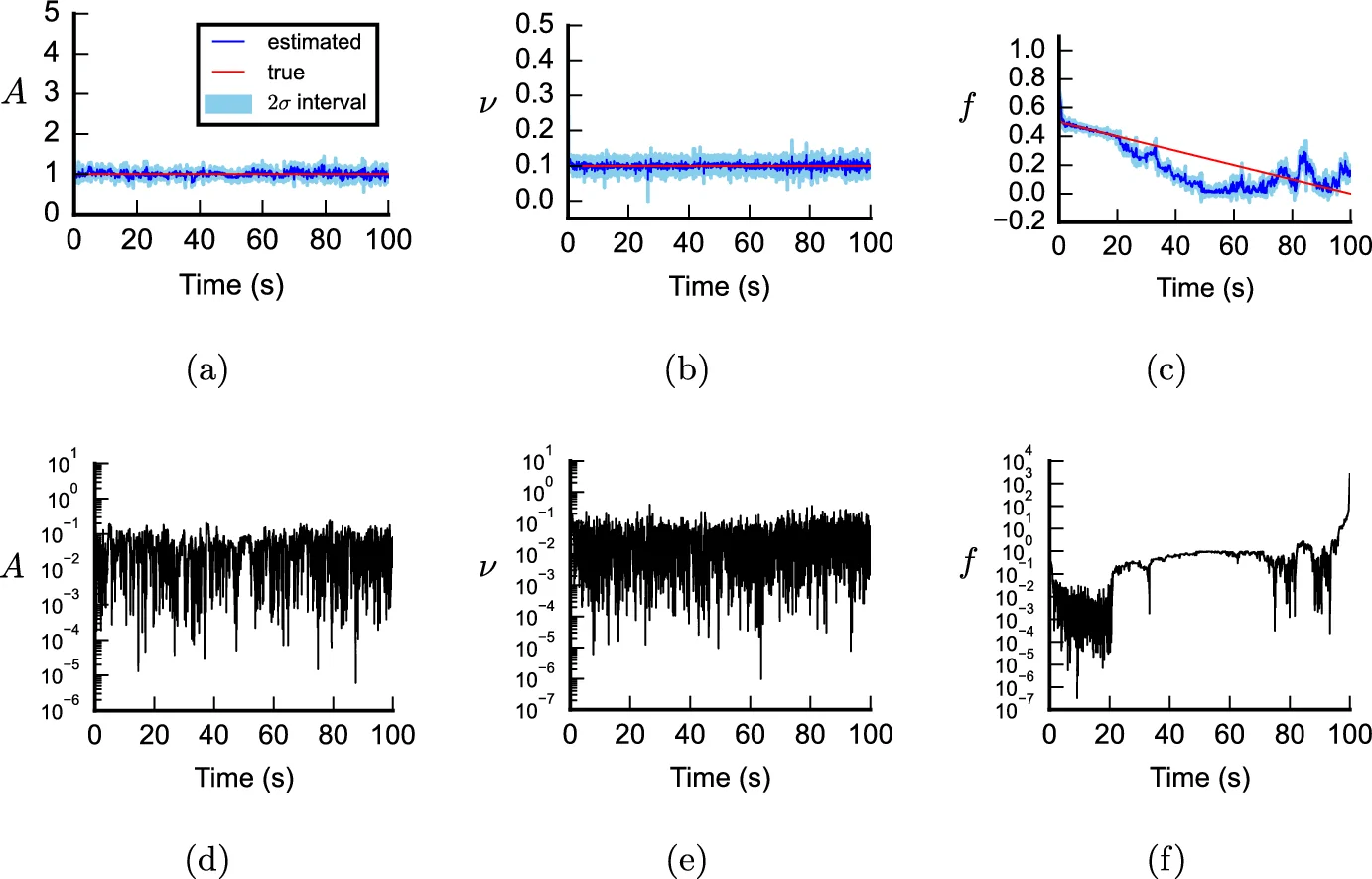
Parameter estimation of the nested particle filter for the data assimilation task with synthetic data, as described in Sect. 3. **a**: The sequence of empirical posterior means $\{A_k\}_k$ (blue) with $\pm 2\sigma$ uncertainty band (shaded region) is compared to the true value $A^*$ (red), showing that the parameter is inferred almost instantly after a very brief burn-in period. **b**: Similar to (a), but for the estimation of the parameter $\nu ^*$. **c**: In the model adopted for inference in (8), the frequency *f* is a parameter, while in the one used for generating the data the frequency is time-dependent ($f^* + a t_k$). The estimated parameter $f_k$ is not constant, as expected, but follows the trend of the time-dependent true variable. **d**–**f**: The relative error for each quantity is plotted as a function of time in a log-linear scale (color figure online)

**Fig. 2:**
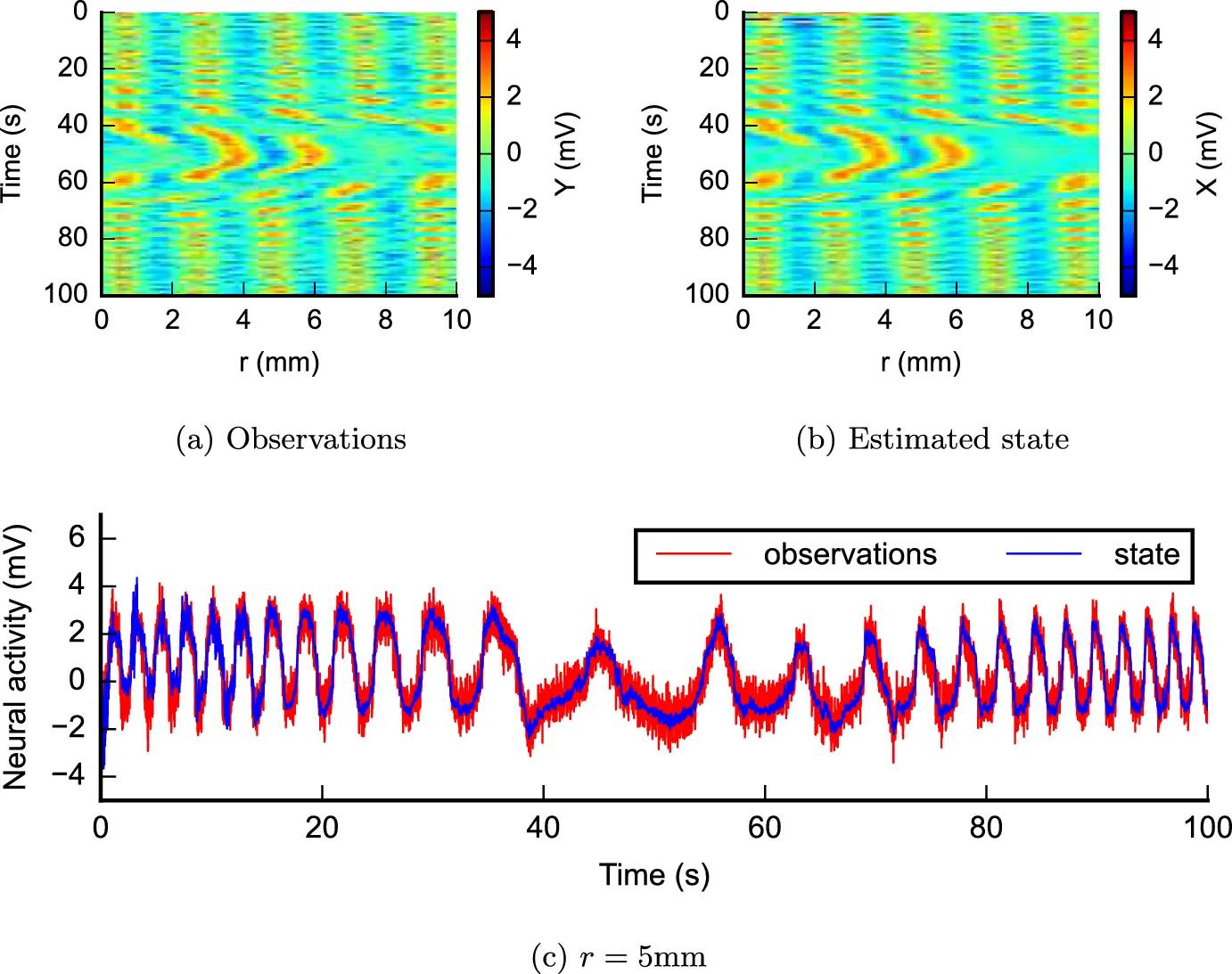
For the same numerical experiment as Fig. 1, we show the observed spatio-temporal data $\{ Y_k \}$ in (**a**), and the estimated state variable $\{ X_k \}$ in (**b**), displaying a good agreement across all spatio-temporal domain. **c**: Time traces at point $r=5\textrm{mm}$ in the cortex, as a function of time

Figure 1a–c show the sequence of empirical posterior means of parameters $\{(A_k, \nu _k, f_k)\}$ and $\pm 2\sigma$ uncertainty band in relation to $\{ (A^*, \nu ^*, f^* + a^* t_k)\}$, while in Fig. 1d–f we examine the time evolution of the relative error in each component. In Fig. 1a and b we observe that the posterior means for the amplitude *A* and spatial frequency ν converge almost immediately after a very brief burn-in period, with a small and stable uncertainty band. This is confirmed by Fig. 1d and e where we see relative errors of the order of $10^{-1}$ for these two parameters. As for the temporal frequency *f*, we observe in Fig. 1c that during the first $20\textrm{s}$, the posterior mean tracks closely the true dynamics, then it progressively diverges below the truth between $20\textrm{s}$ and $50\textrm{s}$, after this, the estimate increases gradually and stays relatively close to the truth. The 2σ bans remains small and stable during the whole observation window.

The particle filter thus estimates correctly parameters that can be meaningfully mapped from the original model $\psi ^*$ to the one used for inference, ψ. A user with knowledge of $\{ Y_k \}$ and $\Psi ^*$, may interpret the lack of convergence in $\{f_k\}$ as model misfit, while in reality this can be used to refine Ψ used in inference. In fact, the model $\Psi ^*$ which gave rise to the observations has a time-dependent frequency *f*(*t*) whose law is not explicitly included in the model Ψ, and the sequence $\{ f_k \}$ tries to approximate it for some time. Through propagation, weighting and resampling of the parameter particles, the filter manages to align the estimated parameter to the observed behaviour of the state dynamics. This can be helpful in refining the state-space model to better align with the observations, by incorporating an additional dynamical component for *f* in Ψ, suggested by the evolution of the sequence $\{f_k\}$.

In Fig. 2 we show the observations (Fig. 2a) and estimated state (Fig. 2b) along with the time evolution in Fig. 2c of both the observations and estimated state at $r=5\textrm{mm}$. In the time series of Fig. 2c, the falling then rising of the temporal frequency of the state, as well as the slowing of the dynamics between $40\textrm{s}$ and $60\textrm{s}$, are plainly visible (see also Fig. 1c).

To assess the state-estimation error over the spatio-temporal grid, we consider the aggregated root mean square error (RMSE)

16 $$\begin{aligned} {{\,\textrm{RMSE}\,}}=\sqrt{\frac{1}{KJ}\sum _{k=1}^{K}\Vert Y_k-X_k\Vert ^2}. \end{aligned}$$

For this synthetic data experiment, we found ${{\,\textrm{RMSE}\,}}=0.561\textrm{mV}$.

**Fig. 3:**
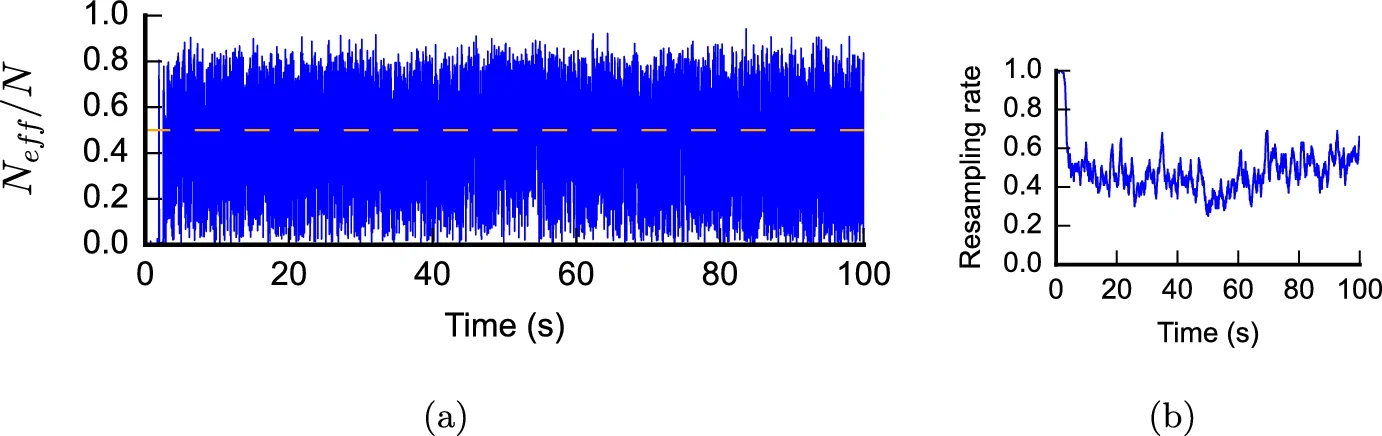
For the same numerical experiment as Fig. 1 we show the sequence of the ratio $N_\text {eff}/N$ in (**a**) and the rolling resampling rate over a window of 100 time steps in (**b**)

To evaluate particle diversity and identify potential degeneracy, we visualize in Fig. 3a the ratio $N_{\text {eff}}/N$ which indicates how many independent samples the parameter particle population is effectively equivalent to. A ratio close to one indicates a well-spread sample, while a value close to zero indicates weight degeneracy and happens when the ensemble collapses. The dashed line indicates the resampling threshold of $N_{\text {eff}}<N/2$. The ratio is near zero in the burn-in period, which may indicate that the initial particle distribution does not match the true parameters. These early estimates are not reliable and should be discarded. After the burn-in period, the filter recovers and oscillates rapidly in the range 0–0.9 with no trend, which indicates frequent resampling. In addition to its role in maintaining sample diversity, this frequent resampling, together with jittering, is a mechanism via which the ensemble of parameter particles moves around in order to account for the dynamics of the true parameters (the frequency *f*) that were not included in the state-space model. This is further confirmed by Fig. 2b, which shows the rolling sampling rate over a window of 100 time steps. At the start, the rate is one which corresponds to the early weight degeneracy discussed before, and indicates resampling at almost each time step during this early stage. After that, the resampling rate settles into a stable regime in the range 0.3–0.7 With a dip in the period 40–60s corresponding to slower dynamics.

While our approach showed its capability to handle joint estimation of (potentially non static) parameters and state of a nonlinear state space model, several limitations need to be pointed out. Particle filter methods suffer from the curse of dimensionality (Snyder et al. 2008; Peter et al. 2008), where the number of particles needed to accurately approximate the posterior distribution grows rapidly with the dimension of the state, often leading to particle degeneracy and increased computational cost. This holds to an even greater extent for the nested particle filter of Algorithm 2, which relies on two layers of filters. Although a 30-dimensional system of the experiment above is not prohibitively large, increasing the state dimension causes rapid growth in particle requirements and computational cost. Additionally, performance is highly sensitive to design choices, most notably the noise intensities *Q* and *R*, the resampling scheme and the jittering kernel (Arulampalam et al. (2002); Doucet and Johansen (2009)). Misspecified *Q* and *R* can result in a collapsed or excessively diffuse ensemble, poor resampling settings can reduce diversity or waste computation, while a jittering kernel with poorly calibrated variance and support can hinder the convergence to the parameter posterior. We also note that discretisation choices when defining the state-space model can introduce further design dependent error. Based on this, the application of such methods requires careful problem-specific tuning to achieve reliable performance. In our case, the noise intensities were chosen by running the algorithm repeatedly over a set of candidate values and visually inspecting the spread of the ensemble of particles and the inferred parameters trajectories; the pair yielding the lowest RMSE while maintaining a high effective sample size was chosen. The discretisation steps were required to allow us to resolve the frequencies present in the observations, and the resampling scheme was chosen based on the results reported in the literature. For additional experiments highlighting the effect of some of these design choices, see Appendices B.1, B.2 and B.3.

## Experimental cortical data

We now make some steps towards performing data assimilation on a recording of the local field potential (LFP) from the cortex of a mouse during natural sleep (Pedrosa et al. 2022), which we take as observations of coarse cortical activity corrupted by noise. In this section we describe preliminary data analysis that was performed prior to data assimilation. We denote the LFP measurements by *V*.

The data provided were labelled into three categories: rapid eye movement (REM) sleep, slow wave sleep (SWS) and unclassified, corresponding to non-specified resting states. The recording has a duration of $T=900\textrm{s}$ with an acquisition rate of $50\textrm{Hz}$, yielding 45000 observations with $\delta t=0.02\textrm{s}$.

**Fig. 4:**
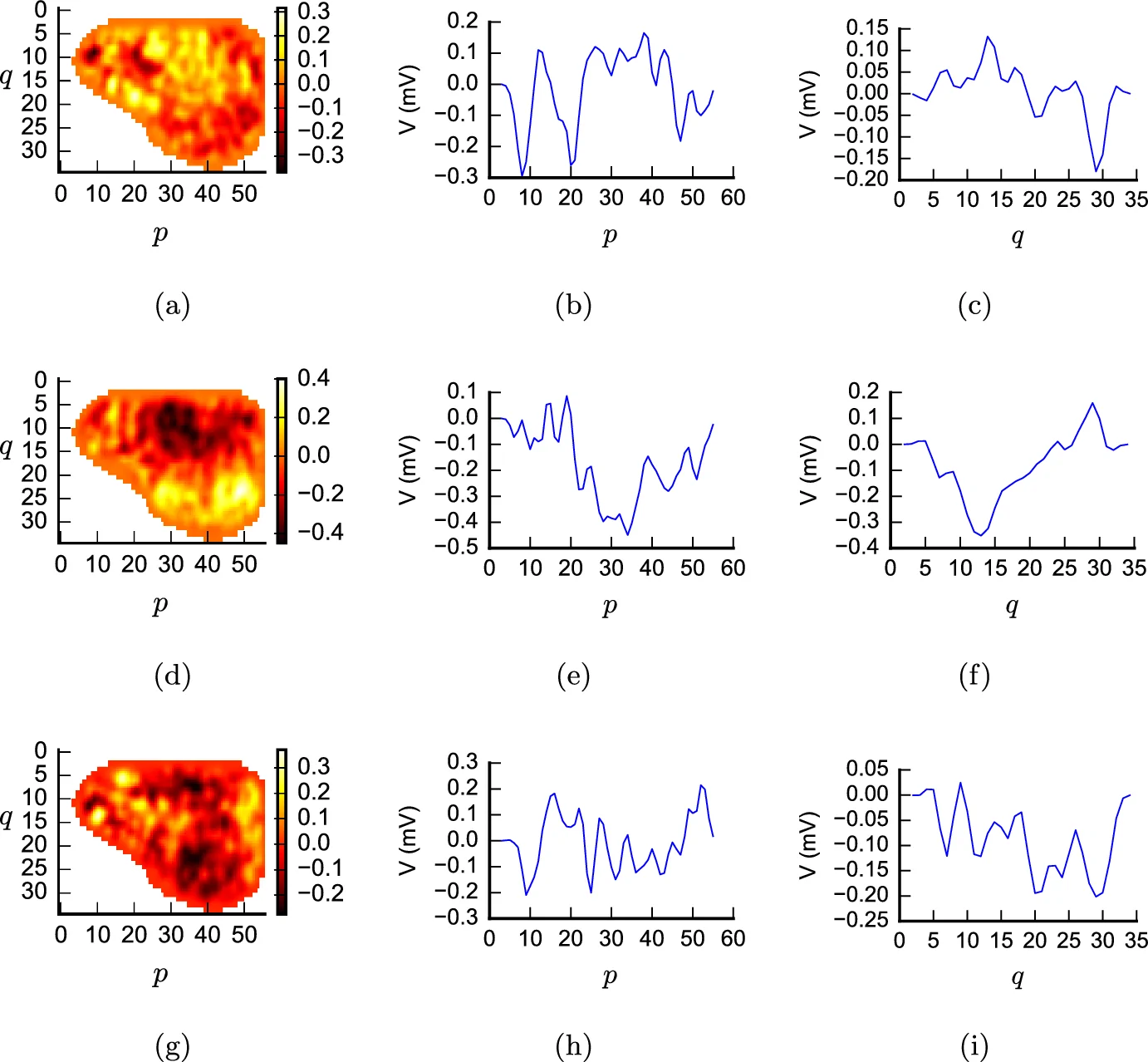
Panels show screenshots from the LFP measurements at three distinct time points corresponding to different sleep stages. Each row represents a different time, progressing from earliest at the top to latest at the bottom: **a**, **b**, **c** are screenshots taken at $t=200\textrm{s}$ during an undefined rest stage, **d**, **e**, **f** are taken at $t=600\textrm{s}$ during slow wave sleep (SWS) and **g**, **h**, **i** at $t=850\textrm{s}$ during rapid eye movement sleep (REM). The columns show three representations in space of the measurements, highlighting the global structure as well as two spatial profiles. By column: the left column **a**, **d**, **g** shows the 2d cortical recordings in the pixel domain (*p*, *q*), the middle column **b**, **e**, **h** a horizontal slice at pixel index q=10 and the right column **c**, **f**, **i** a vertical slice at p=40. This arrangement enables simultaneous visualization of the 2d field along with slices across two principal directions during different sleep stages

In the following, each pixel is indexed by (*p*, *q*), where *p* and *q* are its horizontal and vertical placement in the grid respectively. In Fig. 4 we plot the LFPs from the cortical recording at times t=200, t=600 and t=850 seconds which are data points in the unclassified, SWS and REM sleep stages respectively. Figure 4a, d and g we plot the recordings on the pixel domain (*p*, *q*). We note that at the edge of the recording domain the LFPs is zero. This is further evident in Fig. 4b, c, e, f, h, and i which show the LFPs through a vertical (horizontal) slice at p=40 (q=10). Next we consider the temporal dynamics and dominant frequencies observed in the data before returning to the spatial dynamics.

**Fig. 5:**
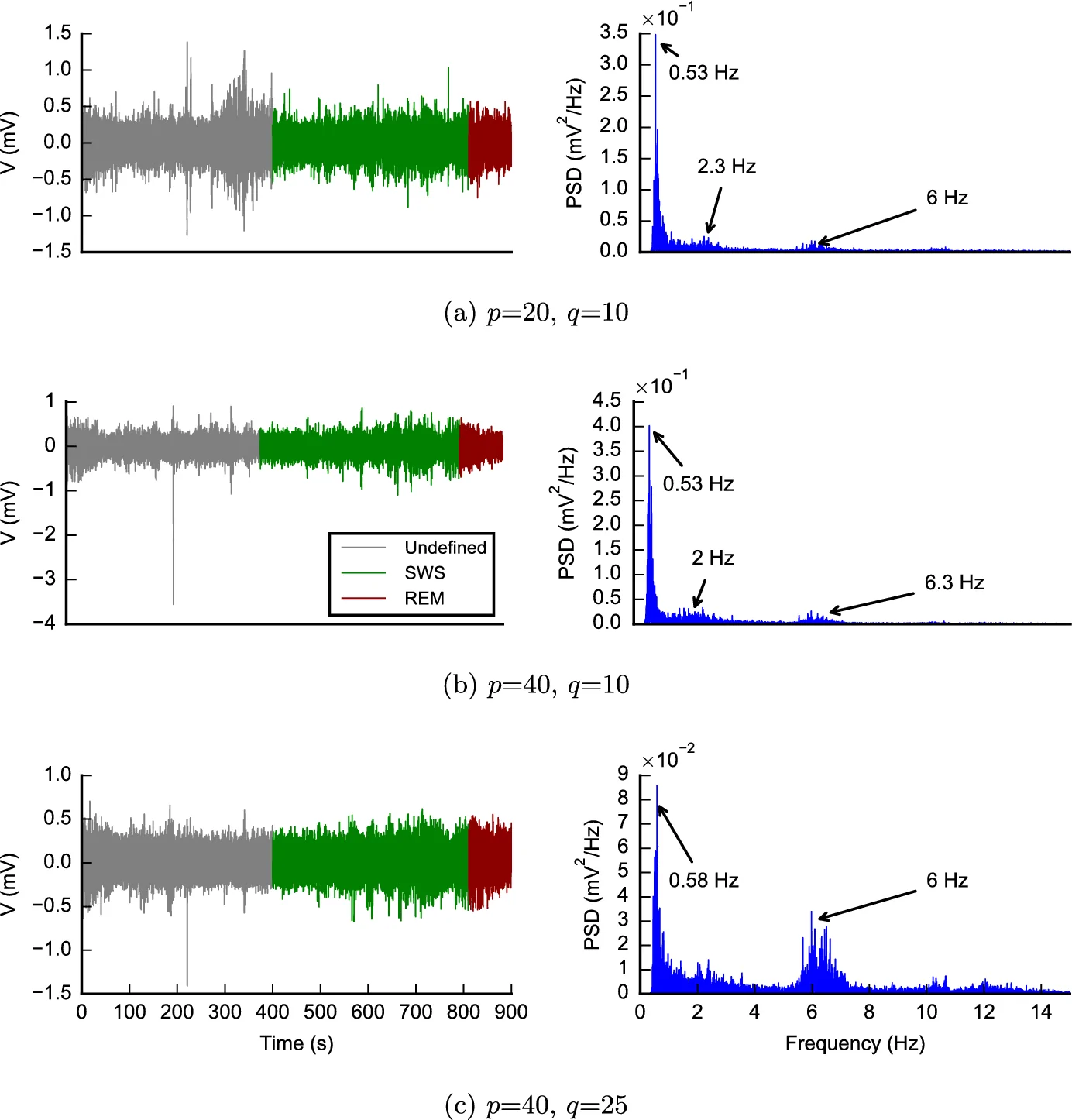
Panels illustrate the temporal dynamics as well as frequency content of three pixels in different locations. Each row shows the time series of the neural activity of a specific pixel on the left, along with its power spectrum density (PSD) and highlighted frequency peaks on the right. **a** corresponds to a pixel on the upper left of the domain (p=20,q=10), **b** corresponds to a pixel on the upper right (p=40,q=10) and **c** to a pixel on the lower right (p=40,q=25). The three panels show similar temporal dynamics between the three pixels, as well as similar frequency profiles with peaks around $0.5\textrm{Hz}$, $2\textrm{Hz}$ and $6\textrm{Hz}$

To illustrate the temporal dynamics we select pixels at different locations in the cortex. These are representative of the kind of temporal dynamics present in the data and are located on the upper left (p=20,q=10), upper right (p=40,q=10) and lower right (p=40,q=25) of the cortex. The time series, along with their power spectrum densities (PSD) computed from a single periodogram, are shown in Fig. 5. We observe a zero-mean oscillatory signal that has a dominant frequency component at $0.5\textrm{Hz}$ and two broad peaks at around $2\textrm{Hz}$ and $6\textrm{Hz}$. The sharp and dominant peak at $0.5\textrm{Hz}$ indicates that energy of the signal is concentrated in the lower frequencies $0-1\textrm{Hz}$, while the broad peaks around $2\textrm{Hz}$ and $6\textrm{Hz}$ may suggest that the underlying process is frequency-modulated or has a frequency that fluctuates over time. The peak at $6\textrm{Hz}$ seems to be more prominent for the pixel located on the lower right of the cortex (p=40,q=25), this may be explained by the higher curvature of the cortex in this region, which introduces high-frequency artifacts in the recordings.

**Fig. 6:**
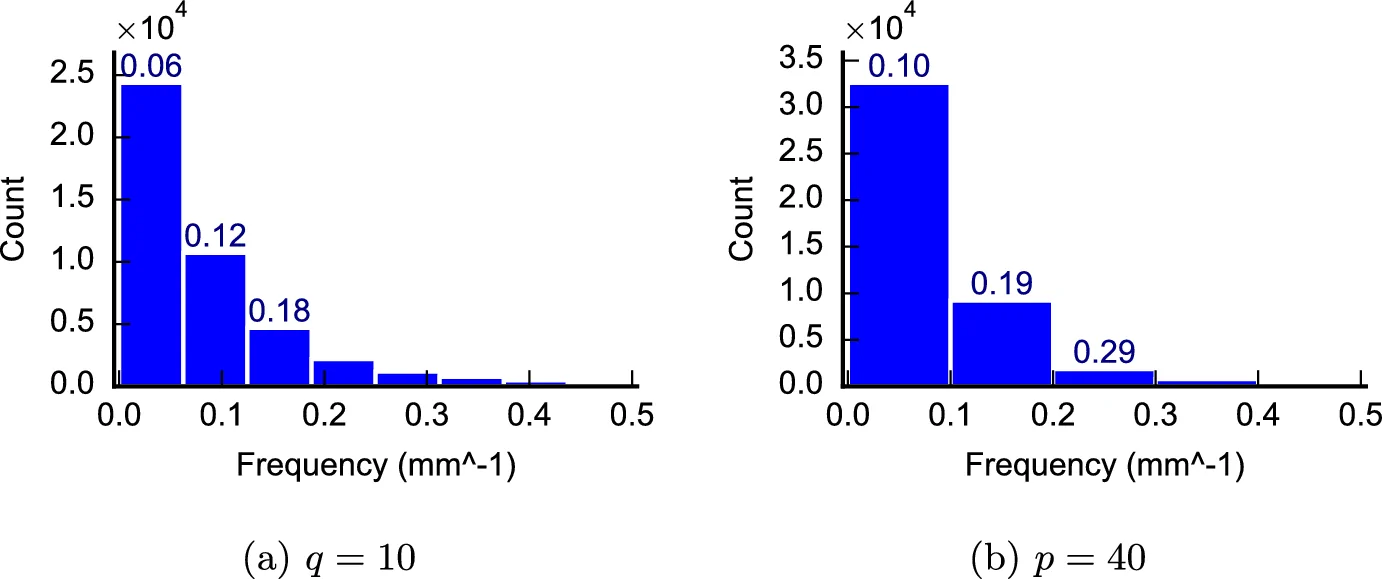
Histograms showing the distribution in time of the dominant spatial frequencies present in two slices at q=10 (**a**) and p=40 (**b**). At each time point, the dominant spatial frequency is extracted by computing the frequency at which the power spectrum density peaks. These peaks are aggregated into a histogram to show their distribution in time. The two histograms exhibit that the temporal evolution for both slices is dominated by larger wavelengths while higher frequency features occur rarely

Next, we consider the spatial frequencies present across the slices at q=10 and p=40. At each time point $t_k$, k=1,…,45000, we extract the dominant frequency by finding the peak of the PSD in space. Figure 6 shows the resulting histograms that aggregate these peaks over the total duration of the recording. In Fig. 6a we examine the horizontal slice at q=10 and in Fig. 6b the vertical slice at p=40. The fundamental frequency for the slice at q=10 is $0.06\textrm{mm}^{-1}$ and $0.1\textrm{mm}^{-1}$ for the one at p=40. Both histograms show a higher concentration of peak spatial frequencies at lower values. This pattern suggests that large scale spatial features (long wavelengths) dominate, while finer scale features (high frequencies) are transient.

### Reduction from two to one spatial dimensions

Since applying the particle filters of Subsection 2.2 requires time-stepping a large number of independent high-dimensional systems of ODEs, we consider reduction from two to one spatial dimension to reduce the computational cost. This enables us to apply the neural mass model of (4) from the discretization of the one-dimensional Amari neural field model (1). To this end we use the Radon transform (Radon 1917) which, as described below, allows us to consider a family of one-dimensional reductions of the dataset, parametrized by an angle α.

To introduce the Radon transform we let $n_{\alpha }=(\cos (\alpha ),\sin (\alpha ))$ be the unit vector with angle α from the *x*-axis. For fixed $d\in \mathbb {R}$ and $\alpha \in [0,\pi )$ we introduce

$$ L_{d,\alpha }=\{(r,s)\in \mathbb {R}^2: \langle (r,s), n_{\alpha } \rangle =d\}, $$

that is, the perpendicular to $n_{\alpha }$ whose distance from the origin is *d*. The Radon transform of a square-integrable function $g :\mathbb {R}^2\rightarrow \mathbb {R}$ is the function $\mathcal {R}g:\mathbb {R}\times [0,\pi ) \mapsto \mathbb {R}$ defined as:

17 $$\begin{aligned} \mathcal {R}g:(d,\alpha ) \mapsto \int _{L_{d,\alpha }} g(z)dS(z)=\int _{\mathbb {R}}\int _{\mathbb {R}}g(r,s)\delta (\langle (r,s), n_{\alpha }\rangle -d)drds. \end{aligned}$$

**Fig. 7:**
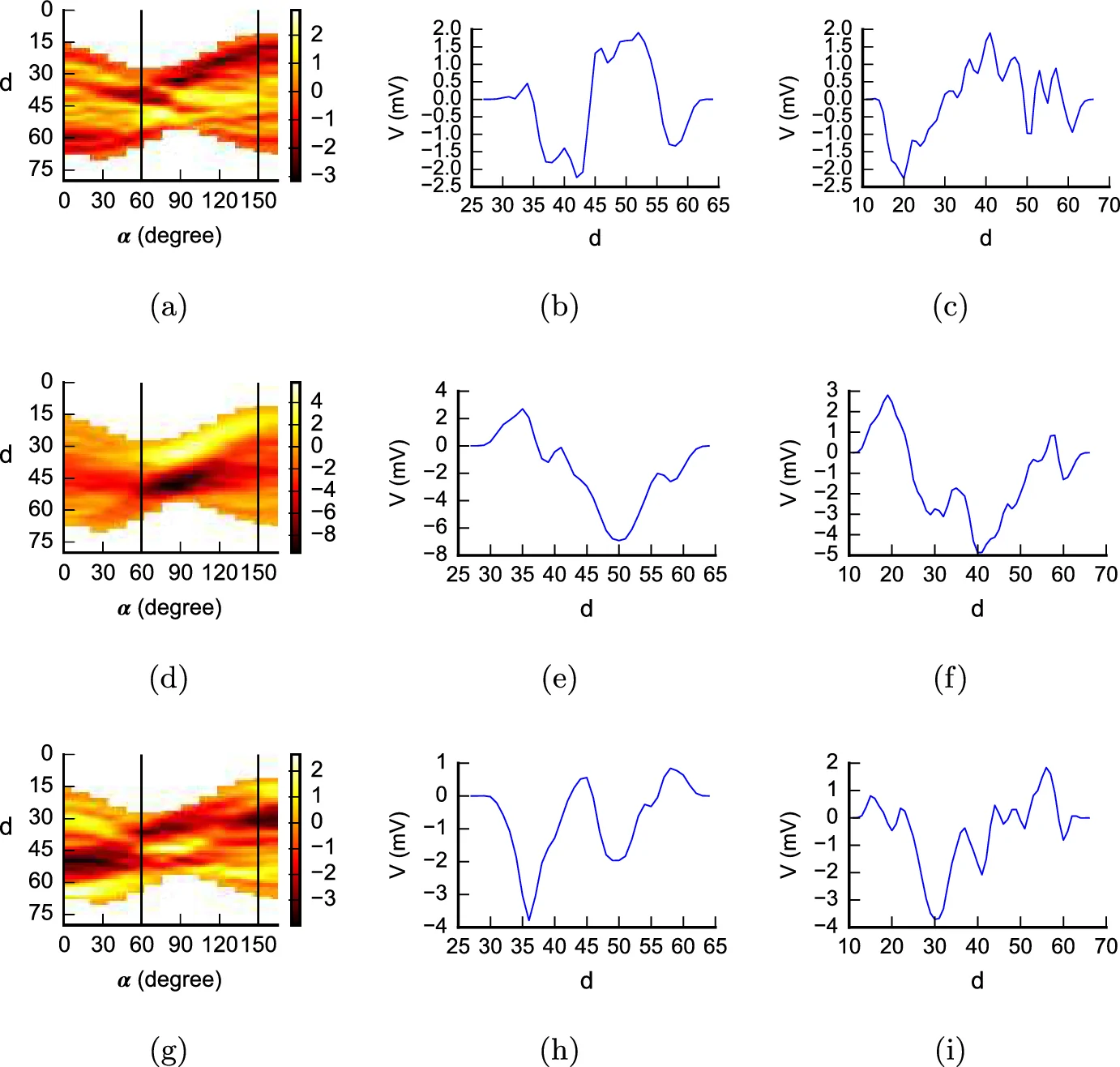
Panels show screenshots from the Radon transform of the LFP measurements at three distinct time points corresponding to different sleep stages. Each row represents a different time, progressing from earliest at the top to latest at the bottom: **a**, **b**, **c** are screenshots taken at $t=200\textrm{s}$ during an undefined rest stage, **d**, **e**, **f** are taken at $t=600\textrm{s}$ during slow wave sleep (SWS) and (**g**, **h**, **i**) at $t=850\textrm{s}$ during rapid eye movement sleep (REM). The columns show three different representations of the measurements in Radon space. The left column **a**, **d**, **g** shows the Radon transform of the 2d measurements in the (d,α) plane, referred to as sinogram. The middle column (**b**, **e**, **h**) shows a slice through the sinograms of the left column at $\alpha =60^\circ$, while the right column (**c**, **f**, **i**) corresponds to a slice at $\alpha =150^\circ$. This arrangement enables visualization during different sleep stages of the full Radon transform along with its spatial profiles for two directions

**Fig. 8:**
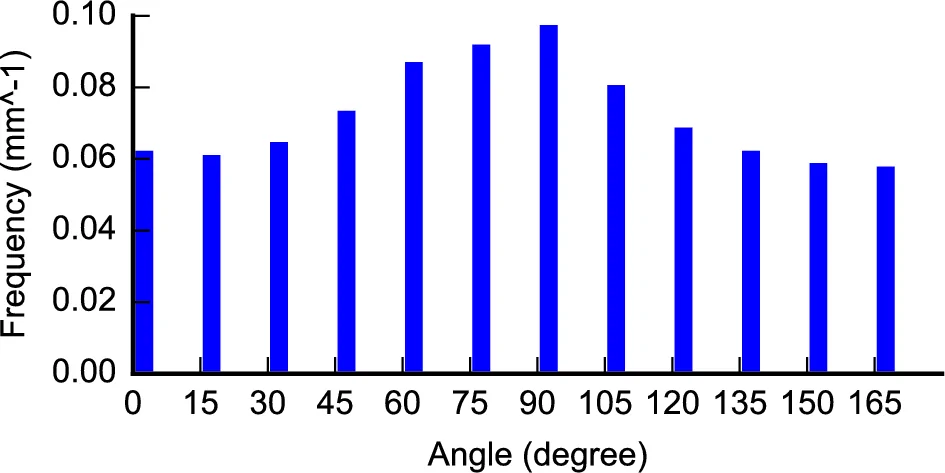
Fundamental spatial frequency in the Radon transformed signal for different angles. Similar to Fig. 6, we construct histograms of the distributions in time of the dominant spatial frequencies present in slices across each angle. Then the most frequently observed frequency of each histogram is taken as the fundamental frequency for that angle

That is the collection of integrals of *g* along the parametrized lines in the plane. Note that in practice the discretized offset samples in the Radon space are given by $$d={\Delta r}(p'\sin (\alpha )+q'\cos (\alpha ))$$, where the indices $p^{\prime },q^{\prime }$ are obtained by centering the pixel domain (*p*, *q*) at the origin (0, 0). For the remainder of this work, we take the Radon domain as our one-dimensional cortical domain and fix $${\Delta r}=0.3\textrm{mm}$$ for all values of α. Our choice of this value is motivated by measurements of the mouse brain reported in the literature (for example (Natt et al. 2002)). In fact, this choice results in spatial domains with lengths ranging from around $$9\textrm{mm}$$ ($$\alpha =90^{\circ }$$) to $$16 \textrm{mm}$$ ($$\alpha =0^{\circ })$$, in agreement with the literature. When $$\mathcal {R}g$$ is visualized in the (d,α) plane (Fig. 7), it typically looks like a superposition of sine and cosine waves and is therefore called a sinogram (Stanley and Deans 1983; Peter Aundal 1996). In Fig. 7 (left), we show the sinogram at different times (and sleep stages) for the data presented earlier in Fig. 4. We also plot slices of the sinogram at angles $$60^{\circ }$$ and $$150^{\circ }$$ that show the reduced 1D spatial signal resulting from the Radon transform at these angles. Compared to Fig. 4 we notice that the 1D signals appear smoother than the original slices of the 2D signals as sharper features are averaged out. We also examine how the fundamental spatial frequency changes with the angle α in Fig. 8. We observe that it changes only slightly with the projection angle, which suggests that the original 2D signal is mostly isotropic and its underlying spatial frequency content does not depend on orientation.

**Fig. 9:**
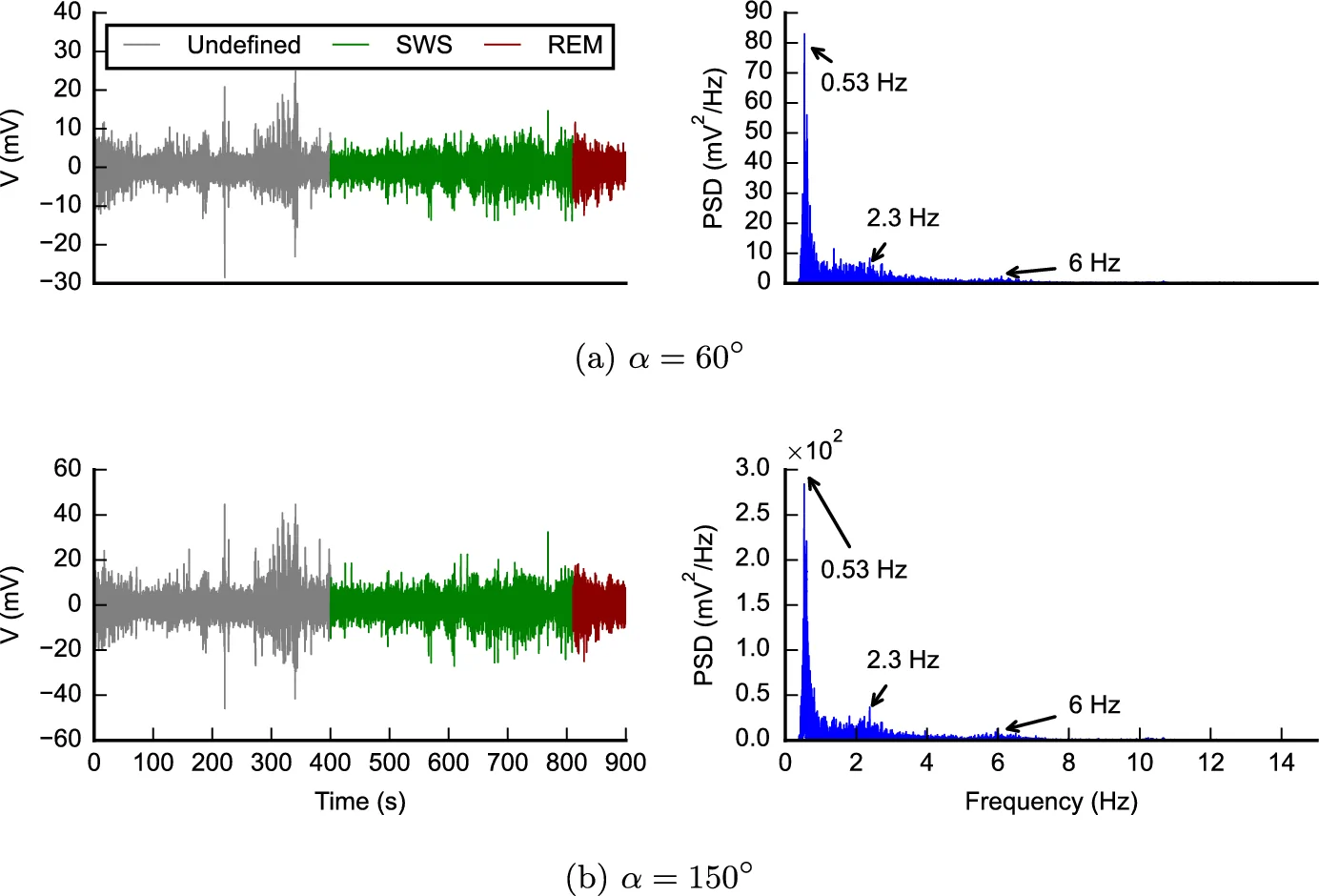
Panels illustrate the temporal dynamics as well as frequency content of the Radon transformed data of the same pixel located at d=40 in the (d,α) plane for two different angles: $60^\circ$ (**a**) and $150^\circ$ (**b**). Each row shows the time series of the transformed signal on the left, along with its power spectrum density (PSD) and highlighted frequency peaks on the right. The panels show similar temporal dynamics and frequency content for the two angles, as well as similar frequency profiles to the original non transformed signal of Fig. 5

For temporal dynamics, Fig. 9 shows the time series and PSD of a spatial node located in the center of the one-dimensional domain for the angles $\alpha =60^{\circ }$ and $150^{\circ }$. By eye, the dynamics appears similar to those observed at pixel level in Fig. 5. Indeed we see that the PSD has the same structure as observed pixel-wise with a dominant peak at around $0.5\textrm{Hz}$ and two broad peaks around $2\textrm{Hz}$ and $6\textrm{Hz}$.

We conclude that using the Radon transform to reduce data from two to one dimension preserves important features in terms of spatial and temporal frequencies. In our investigation below, we will perform data assimilation for different angles α.

## Nested particle filter for the Radon transformed cortical data

### Results

We now move to the inference of parameters and state of the discrete model (8), with external input parametrised as in Sect. 3.

In Fig. 7, we observed that the signal tends to zero at the edges of the domain, and system (8) can reproduce this behaviour with a specific choice of the observation matrix *G*. Here we select $G = {\textbf {T}}(J,\beta )$ where ${\textbf {T}}$ is a *Tukey window*, also known as *cosine-tapered window* (Harris 1978), with parameters (J,β). For a vector of size *J* and a tapering fraction $\beta \in [0,1]$, ${\textbf {T}}(J,\beta )$ is a diagonal *J*-by-*J* matrix acting as a mask: the multiplication ${\textbf {T}}(J,\beta )v$ tapers the first and last β/2 components of *v* using a cosine function, and leaves the rest intact. The diagonal of ${\textbf {T}}$ is given by

18 $$\begin{aligned} {\textbf {T}}_{jj}=\left\{ \begin{array}{lcr} \frac{1}{2}\left[ 1+\cos {\left( \pi \left( \frac{2j}{\beta (J-1)}-1\right) \right) }\right] & \text{ for } & 0 \le j< J_\ell , \\ 1 & \text{ for } & J_\ell \le j \le J_r, \\ \frac{1}{2}\left[ 1+\cos {\left( \pi \left( \frac{2j}{\beta (J-1)}-\frac{2}{\beta }+1\right) \right) }\right] & \text{ for } & J_r<j\le J-1, \end{array}\right. \end{aligned}$$

where $J_\ell$ and $J_r$ are the number of samples in the left and right tapered region respectively, given by

$$ J_\ell =\left\lfloor \frac{\beta (J-1)}{2}\right\rfloor ,\quad J_r=J-1-J_\ell . $$

In our case, we fix the tapering parameter β=0.5. The state-space model in use is then (8) in which $\Psi (X,\theta )$ is defined with a forcing I(t;θ,0) with components $\left( \Xi (r_j,t,\theta ,0)\right) _{j=0}^{J-1}$

$$ \Xi (r,t,\theta ,0) = A \cos 2 \pi \bigl (\nu r - ft\bigr ), $$

and for which

$$ G = {\textbf {T}}(J,0.5),\qquad Q = R = 0.5, \qquad \eta _k, \gamma _k {\mathop {\sim }\limits ^{i.i.d}} \mathcal {N}(0,{{\,\textrm{Id}\,}}_J), \qquad k = 1,\ldots ,K. $$

We run the nested particle filter, Algorithm 2, with N=M=250 particles, K=45000 time steps, $\delta t=0.02\textrm{s}$, uniform priors on parameters

$$ (A,\nu ,f)\sim \mathcal {U}([0,30])\otimes \mathcal {U}([0,1])\otimes \mathcal {U}([-10,10]), $$

and Gaussian prior on the state $X_0$, as given in (15). Note that the size *J* of the state vector changes depending on the Radon angle α, and δt is the inverse of the sampling rate of the LFP measurements. The tuning parameters, *Q*, *R*, β and the prior ranges were chosen from a set of candidates consistent with the characteristics of the observations ( amplitude and frequencies), for which we run Algorithm 2 and monitor the behaviour of variance, *RMSE* and effective sample size. For some synthetic experiments illustrating the effect of misspecification of *Q*, *R* and the prior ranges, we refer the reader to Appendices B.2 and B.3. We also reproduce the same experiments described in this section for two additional values of the tapering fraction β in Appendix B.4.

**Fig. 10:**
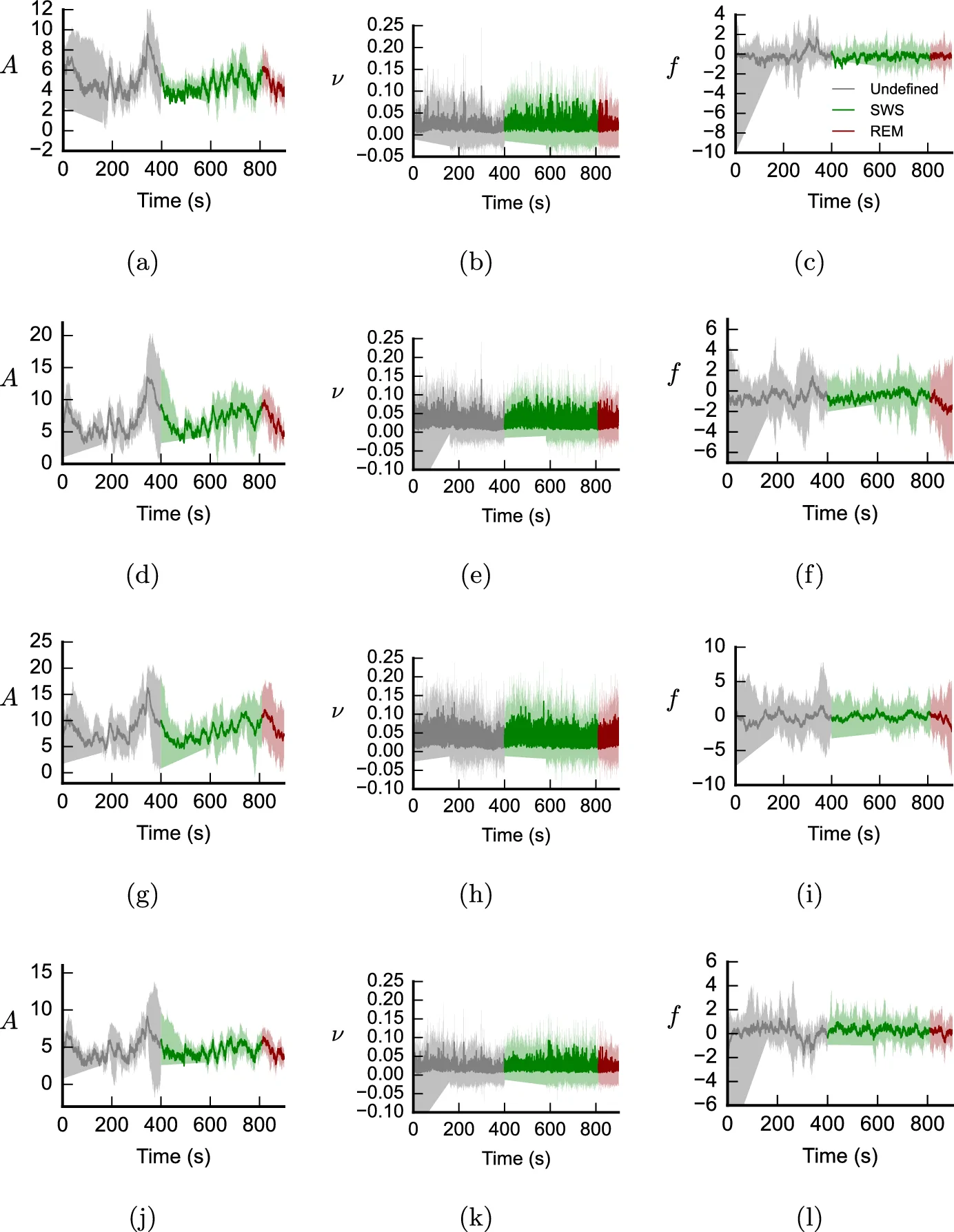
Evolution in time of the posterior mean estimate of the input parameters for different angles α with $\pm 2\sigma$ band. Each panel displays the average over 10 independent runs of the nested particle filter of Algorithm 2. Columns, from left to right, correspond to parameters of the forcing: amplitude *A*, spatial frequency ν and temporal frequency *f*. Rows, from top to bottom, correspond to Radon angles $0^{\circ }$, $60^{\circ }$, $90^{\circ }$ and $150^{\circ }$. The first 20 time steps are excluded in order to remove the burn-in period

We consider the one-dimensional observations obtained from the Radon transform with angles $\alpha =0^{\circ },60^{\circ },90^{\circ },150^{\circ }$, as described in Subsection 4.1. We select this subset of angles because it spans the dominant frequency behaviours exhibited in Fig. 8 without redundancy. Moreover, orthogonality of the two pairs ensures that each selected projection angle contributes maximally independent information about the original 2D signal. In Fig. 10 we show the evolution over time of the empirical mean estimates of the parameters *A*, ν and *f* for the average of ten independent runs of Algorithm 2. Note that the posterior mean estimates of the parameters (A,ν,f) were initialized with (15, 0.5, 0), however, these values are not shown on Fig. 10 because the first 20 time steps (burn-in) were discarded for clarity. We also show the 2σ band around the mean estimates, where σ is the total standard deviation that combines the average within-run and between-run variances, such that

19 $$\begin{aligned} \sigma ^2= \frac{1}{10}\sum _{i=1}^{10}\sigma _i^2 + \frac{1}{10}\sum _{i=1}^{10} (\mu _i-\bar{\mu })^2, \end{aligned}$$

where $\sigma _i^2$ and $\mu _i$ are the empirical variance and posterior mean for run *i*, and $\bar{\mu }=\frac{1}{10}\sum _{i=1}^{10} \mu _i$ is the aggregated posterior mean across runs.

Across the four Radon angles, the posterior mean of parameter ν converges rapidly from its initialization value of 0.5 to a value between 0 and 0.15, with no major differences between sleep stages. The estimates for angles $60^\circ$ and $90^\circ$ (Fig. 10e and h) are slightly more elevated than those for angles $0^\circ$ and $150^\circ$ (Fig. 10b–k). This is consistent with the spatial scales present in the observations (Fig. 8). The 2σ band is tight and consistent across angles and sleep stages. This supports the conclusion that the observed cortical patterns can be produced by a neural mass model with external forcing with spatial frequencies ν in the range $0-0.15\textrm{mm}^{-1}$.

**Fig. 11:**
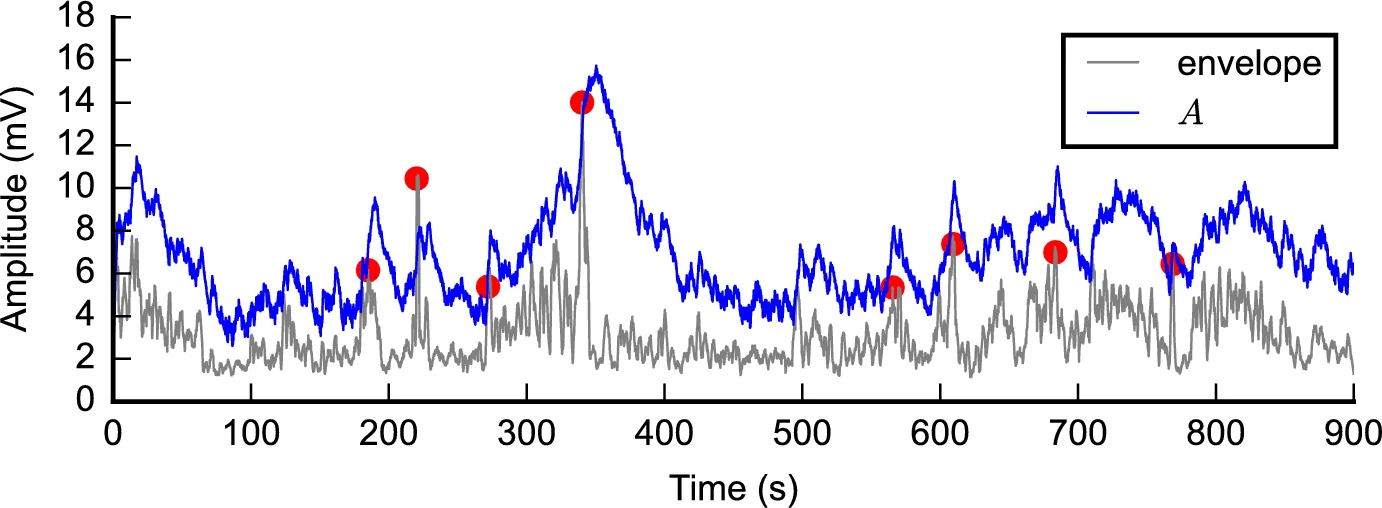
Posterior mean of *A* and envelope of observed signal at $r=5.4\textrm{mm}$ for $\alpha =60^{\circ }.$

In contrast, *A* and *f* exhibit a more oscillatory behaviour. From an initialization of 15, the posterior mean estimate of *A* settles, to a first approximation, into oscillatory phases type $A(t) = \bar{A} + \tilde{A}(t)$. We note that $\bar{A}$ changes value across Radon angles, while $\tilde{A}(t)$ seems to have comparable temporal frequency. We also see abrupt changes in *A*(*t*) at the onset of the slow wave sleep (SWS) episode.

In particular, the magnitudes $\bar{A}$ for angles $60^{\circ }$ and $90^{\circ }$ (Fig. 10d and g) are higher than those for angles $0^{\circ }$ and $150^{\circ }$ (Fig. 10a and j). This is expected since the horizontal line integrals that correspond to the radon transform for $\alpha =90^{\circ }$ have more pixels than the vertical ones, and therefore receive on average more input. For $\alpha =0^\circ$, the filter starts with a wide 2σ band that narrows as the filter accumulates information, without a noticeable difference between sleep stages. For the other angles, the 2σ band widens between $300\textrm{s}$ and $500\textrm{s}$, which corresponds to the transition from the undefined sleep stage to SWS. For the rest of the observation window, the 2σ band remains moderate. Moreover, the global time trace *A*(*t*) captures the dynamics of the amplitude of the observations as illustrated in Fig. 11. The figure displays the posterior mean of *A*(*t*) averaged over ten runs for $\alpha =60^{\circ }$ and the envelope of the one-dimensional observations at $r=5.4\textrm{mm}$ for the same angle. The envelope was extracted using a moving root mean square with window length of 100 discrete time points corresponding to $20\textrm{s}$. Figure 11 shows how *A* is closely linked to the dynamics of the envelope and how the peaks of the two match, supporting the conclusion that our choice of the forcing I(t;θ,0) is suitable to act as a forcing term to the observed neural activity patterns.

The red discs indicate peaks of the envelope that exhibit gradual rise and rapid decay in the cortical signal. These abrupt jumps cause a lag in the estimation of *A*, reflecting the efforts of the ensemble of particles to adapt to the new unexpected observation. This is particularly apparent for the peak around $340\textrm{s}$, where the envelope changes rapidly from around $18\textrm{mV}$–5. It takes the filter about $60\textrm{s}$ to track this change. In contrast, the gradual changes are tracked with little lag, see for example the periods between $100\textrm{s}$ and $150\textrm{s}$, and then $450\textrm{s}$ and $550\textrm{s}$. Additionally, we find that both the envelope and *A* have a main oscillatory component of $\tilde{A}(t)$ at frequency $0.002\textrm{Hz}$. Returning to Fig. 10, we observe an oscillatory behaviour that depends on the angle for the temporal frequency parameter *f*. These oscillations appear especially during the undefined rest and REM sleep, while *f* appears to settle near $0\textrm{Hz}$ (Fig. 10c, i and l) and $-0.5\textrm{Hz}$ (Fig. 10f) during SWS sleep. We observe also a change in sign which, in view of the input parametrisation $\Xi (r,t;A,\nu ,f,0) = A \cos 2\pi (\nu r - f t)$, indicates a change in the direction of wave propagation from left to right. We note also the occurrence of a burst in *A* and *f* simultaneously around $340\textrm{s}$, before the onset of slow wave sleep. As for the uncertainty, estimates for angles $0^\circ$ and $150^\circ$ exhibit a moderate and stable 2σ with few spikes across the different sleep stages. In contrast, the uncertainty bands for $\alpha =60^\circ$ and $\alpha =90^\circ$ are initially large for the first $100\textrm{s}$, narrower until around $300\textrm{s}$ when they spike before the transition to SWS, during which they stay stable and moderate, then they widen sharply again towards the end during REM sleep. This abrupt re-expansion of uncertainty at the regime transition, exhibited also for parameter *A* (Fig. 10d, g, and j), suggests the filter is struggling to adapt to a changes in the dynamics.

Next, we are interested in aggregating the estimated posterior means of *A* and ν into time blocks and approximating their distributions within each block. This enables us to examine the global statistical structure of these estimates, particularly how they spread and cluster, rather than the instantaneous fluctuations from the time traces in Fig. 10. For this purpose, we split the estimates into blocks of 5000 discrete time points, then approximate the density of the distribution of each block. These densities are estimated using a standard kernel density estimation method with a Gaussian kernel $K_h$ of bandwidth *h* approximated using Silverman’s rule (Silverman 1986): $h=0.9\overline{\sigma }n^{ -1/5}$, where n=5000 is the sample size of each block and $\overline{\sigma }$ is the sample variance.

**Fig. 12:**
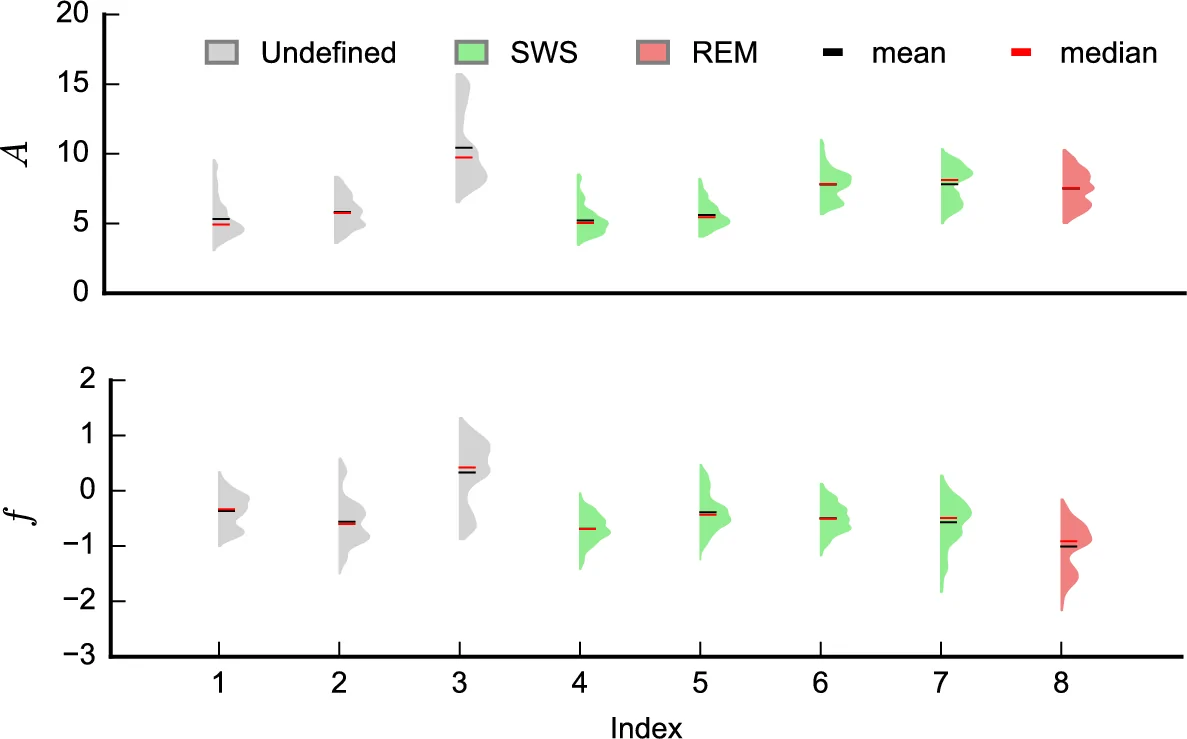
Estimated density of equal blocks of the posterior mean estimates of input parameters *A* (top) and *f* (bottom) for angle $\alpha =60^{\circ }$. Each panel displays the kernel density estimation of blocks of 5000 discrete time points of the average over 10 independent runs of the nested particle filter of Algorithm 2. In each panel, index i=1,⋯,8 indicates the block between 5000*i* and 5000(i+1)

Figure 12 shows the densities of *A* and *f* for $\alpha =60^{\circ }$. We observe that for both parameters *A* and *f*, the densities are multimodal and that the central tendencies, captured by the mean and median, as well as the shape of the density change between blocks. This is in line with the non-stationarity of the amplitude and temporal frequency of the observations. We observe a sudden jump for the mean and an increase in variability in the third block preceding the SWS between $300\textrm{s}$ and $400\textrm{s}$. During SWS, blocks four and five for *A* have similar shapes and their mean and median do not change much (and similarly for blocks six and seven). As for *f*, blocks four to seven have a mean around $-0.5\textrm{Hz}$ and their shapes change smoothly. We also notice the appearance of a tail in the lower values of *f* (between $-1.5\textrm{Hz}$ and $-2\textrm{Hz}$) for block seven, which transforms into a new cluster in that frequency band in block eight during REM. The range of the distribution of *f* is consistent with the temporal frequency analysis of the observation (Fig. 9), which revealed the presence of two peaks around $0.5\textrm{Hz}$ and $2\textrm{Hz}$.

We conclude from the examination of the inferred input parameters that our model (Eq. (8)) can handle the challenges posed by the LFP measurements. This consists in modelling the forcing as a travelling space-time wave with three parameters *A*, ν and *f* that relate to concrete features of the LFP measurements. In fact, *A* was shown to track the dynamics of the envelope of the observations, ν the spatial frequencies and *f* the temporal frequencies. The mean posterior estimates of these parameters were consistent with the scales naturally present in the observations, and they were able to flexibly track the non-stationary behaviour of these features. Moreover, the transition from undefined rest state to slow wave sleep was demonstrated by the simultaneous occurrence of a sharp spike in both *A* and *f*. During SWS, the parameters were shown to change gradually, and the magnitudes of the jumps, if present, were generally smaller than during the other sleep stages. Additionally, the absence of significant variations by projection angle indicates that a spatially uniform input or a superposition of waves with different and even time varying propagation directions could be a suitable model for the input when considering the original two-dimensional LFP measurements.

**Fig. 13:**
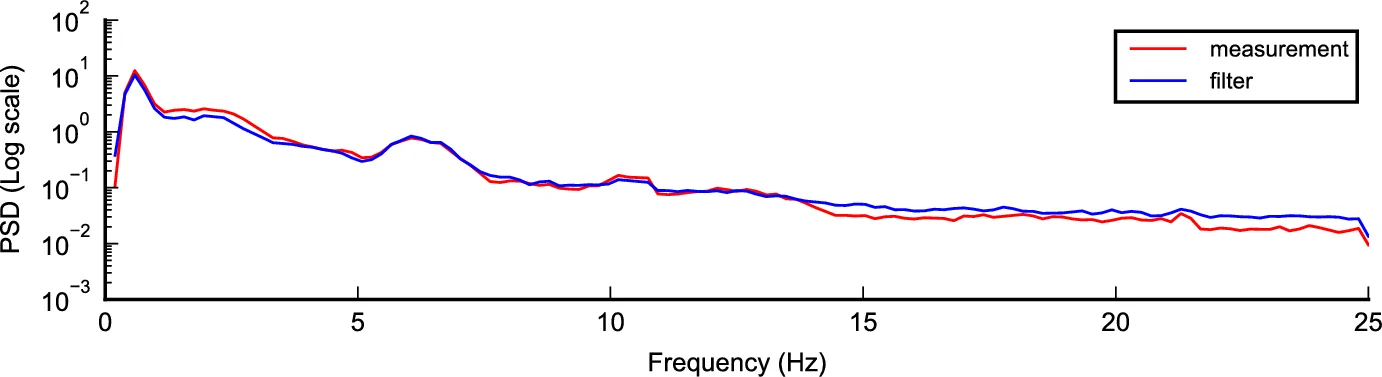
Temporal PSD of estimated state and observations at $x=4.2\textrm{mm}$ for $\alpha =60^{\circ }$

Next, we shift our focus to state estimation. To assess its quality, we take as our metric the aggregated ${{\,\textrm{RMSE}\,}}$ as defined in Eq.  (16). In Table 2 we show the averaged ${{\,\textrm{RMSE}\,}}$ over the ten runs of Algorithm 2 by angle. The values reported indicate good state reconstruction and we notice no great difference between the four angles (with only $90^{\circ }$ having a slightly higher error). In addition, we also compare the PSDs of the observed signal and the estimated state at a single spatial node for illustration. Figure 13 shows the two PSDs, calculated using Welch’s method (Welch 1967), on a linear-log scale at $r=4.2\textrm{mm}$ for $\alpha =60^{\circ }$ in Fig. 13. We opted for this method instead of the single periodograms shown in the previous sections, because it provides reduced variance and smoother estimates that are easy to examine visually. We observe identical overall frequency structures, with peaks aligned in the dominant frequency bands around $0.53\textrm{Hz}$, $2\textrm{Hz}$ and $6\textrm{Hz}$. Furthermore, since the frequency peak at $0.53\textrm{Hz}$ corresponds to the maximum signal energy in both cases, we can calculate the deviation of the value of this frequency peak between the observed and estimated state across the whole spatial domain. We denote this quantity by $\Delta f_t$, so that

20 $$\begin{aligned} \Delta f_t:=\frac{1}{J}\sum _{j=0}^{J-1} \left| \arg \max _{\omega \in \Omega }|\mathcal {F}(Y(r_j,t))(\omega )|^2-\arg \max _{\omega \in \Omega }|\mathcal {F}(X(r_j,t))(\omega )|^2\right| , \end{aligned}$$

where $\mathcal {F}$ is the Fourier transform and Ω is the frequency domain. The average of this metric over ten independent runs is given in Table 2. This shows a low deviation across the four angles, which indicates the conservation of the main frequency in time. Our approach of joint parameter and state estimation is successful at preserving the dominant oscillatory components of the LFP measurements, which highlights its ability to accurately capture meaningful dynamics while learning the parameters of the model that was proposed to have generated them.

**Table 2:** Errors made in state estimation for different Radon angles

Angle (degree)	${{\,\textrm{RMSE}\,}}$ ($\textrm{mV}$)	$\Delta f_t$ ($\textrm{Hz}$)
0	1.6565	0.0080
60	1.9295	0.0409
90	2.4985	0.0222
150	1.5523	0.0331

## Conclusion

This work presents a proof-of-concept effort to jointly estimate the state and parameters of a neurobiological rate network model from LFP measurements of a mouse cortex during natural sleep. For this purpose, we used a nested particle filter on one-dimensional observations obtained by the Radon transform of the original, two-dimensional measurements.

Initially, we tested this approach on synthetic data, and showed that the algorithm correctly estimates states and learns parameters, including those that vary in time following dynamics not explicitly included in the model. This held true for the cortical data as well; we were able to successfully track the observations while preserving their main frequency characteristics through the different sleep stages and for different projection angles in the one-dimensional reduction.

Our choice of the Amari neural field model was motivated mainly by its being one of the simplest integro-differential equations that capture the nonlinear and nonlocal nature of the interactions between the neurons. Moreover, there is an abundance of results about existence and uniqueness of the solutions and their properties. However, the model assumes a homogeneous neuronal population and parameters, which is unrealistic for actual cortical systems. We also chose the simplest of discretization schemes in order to avoid unnecessary computational overhead or the need to estimate additional parameters for more complex schemes. Furthermore, such methods work well with acceptable precision for smooth solutions, as is the case for the neural field. Our goal in this work was not to achieve optimal modeling performance, but rather to demonstrate the feasibility and potential of Bayesian estimation based on neural mass models for LFP cortical measurements. These initial findings motivate future work on refining the state-space models and on parameter estimation for cortical data. This includes using multiple heterogeneous populations of excitatory-inhibitory neurons, or models that can be more directly mapped to microscopical neural activity.

## Figures and Tables

**Fig. 14:**
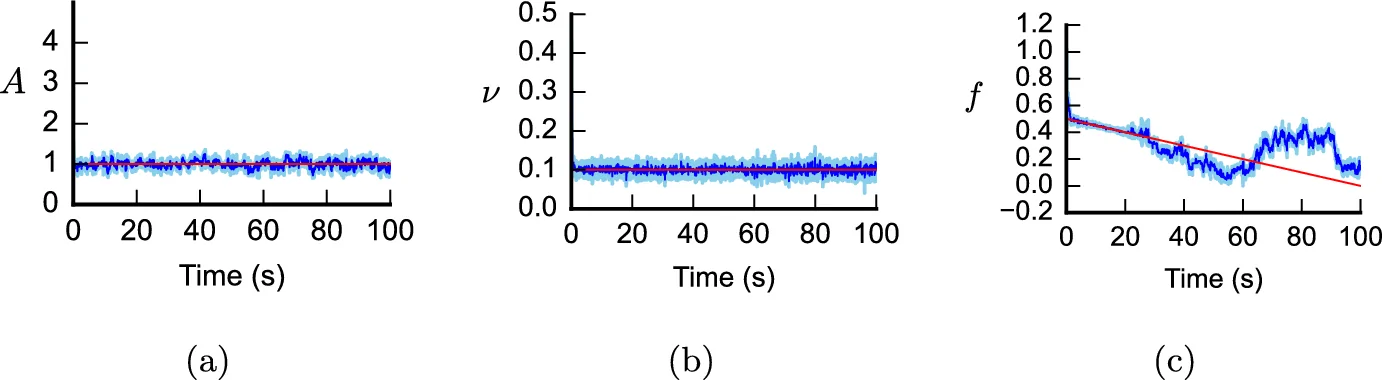
$$J=30,\;\Delta t=0.02$$

**Fig. 15:**
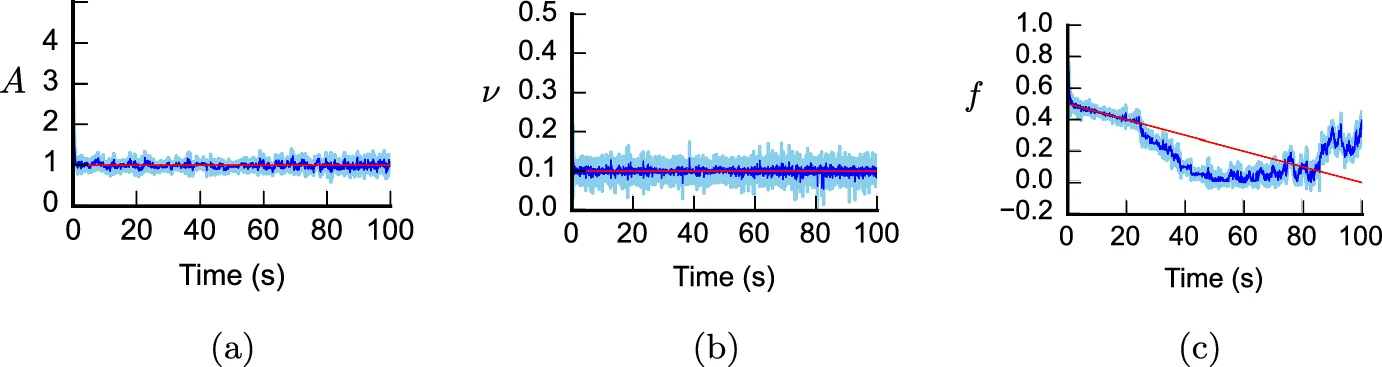
$$J=30,\;\Delta t=0.005$$

**Fig. 16:**
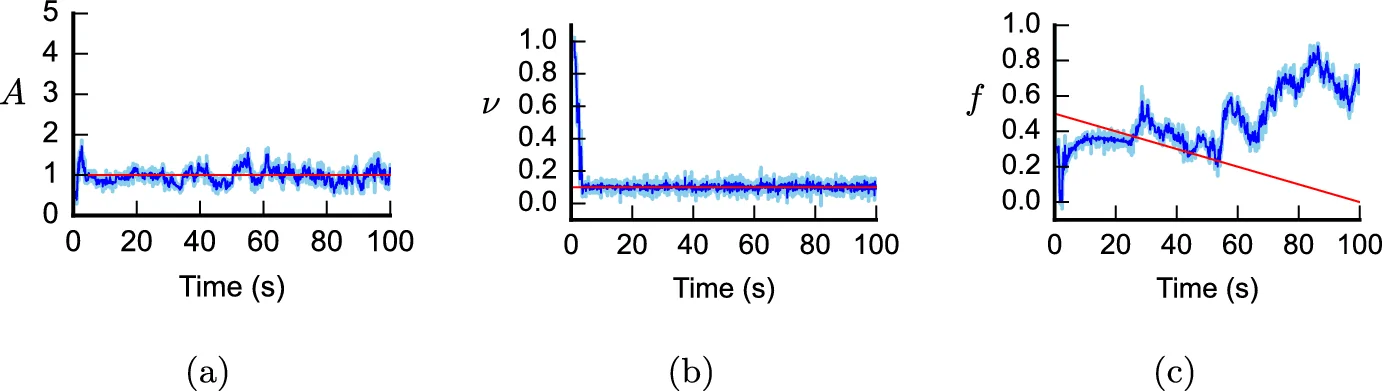
$$J=15,\; \Delta t=0.01$$

**Fig. 17:**
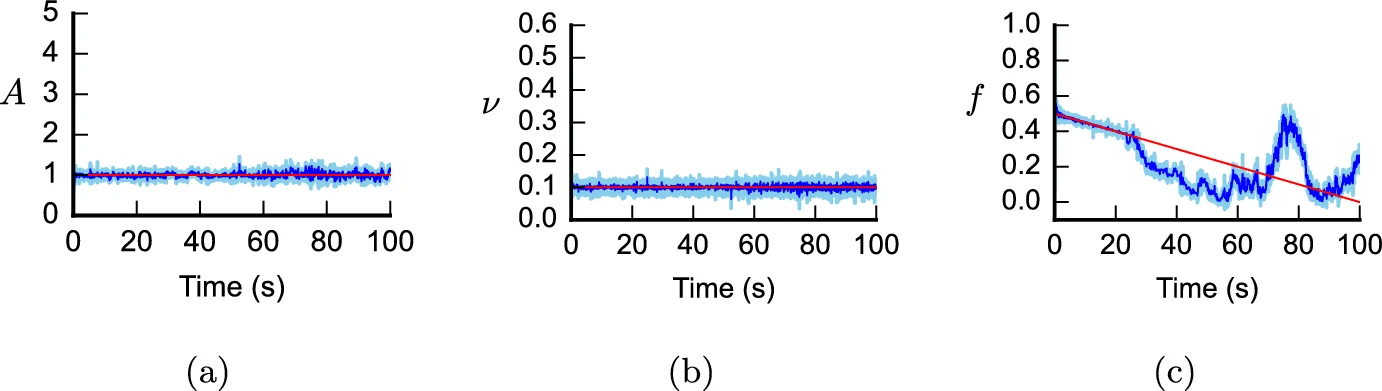
$$J=45,\; \Delta t=0.01$$

**Fig. 18:**
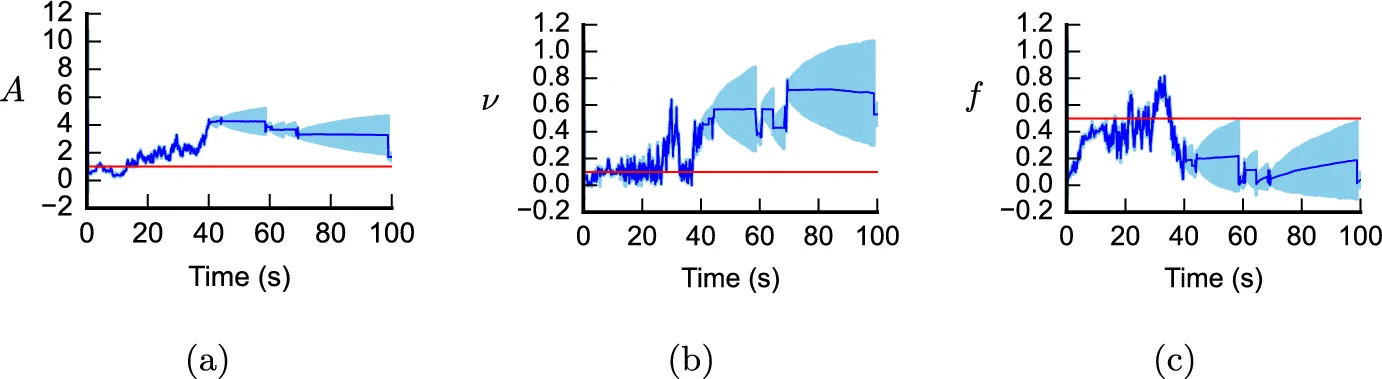
q=0.1,R=0.1

**Fig. 19:**
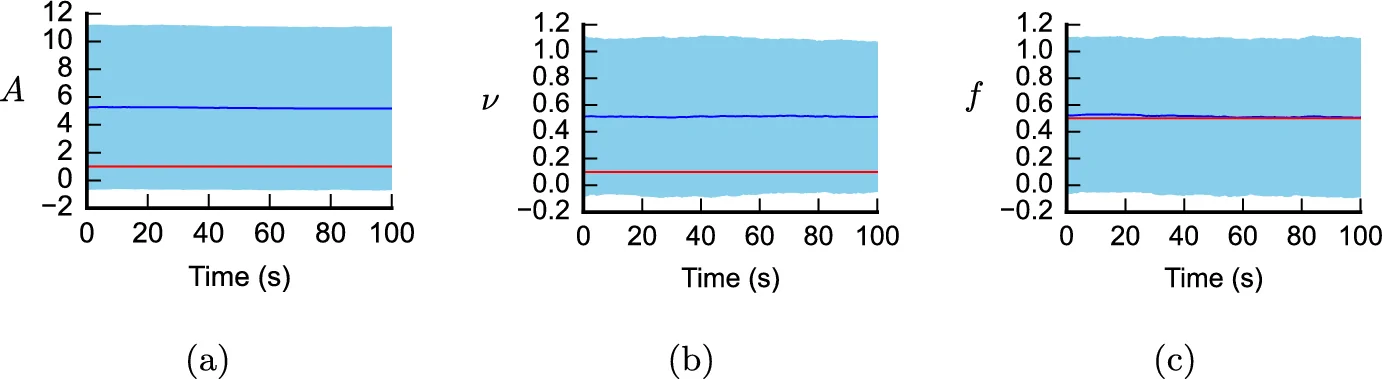
q=0.5, R=0.1

**Fig. 20:**
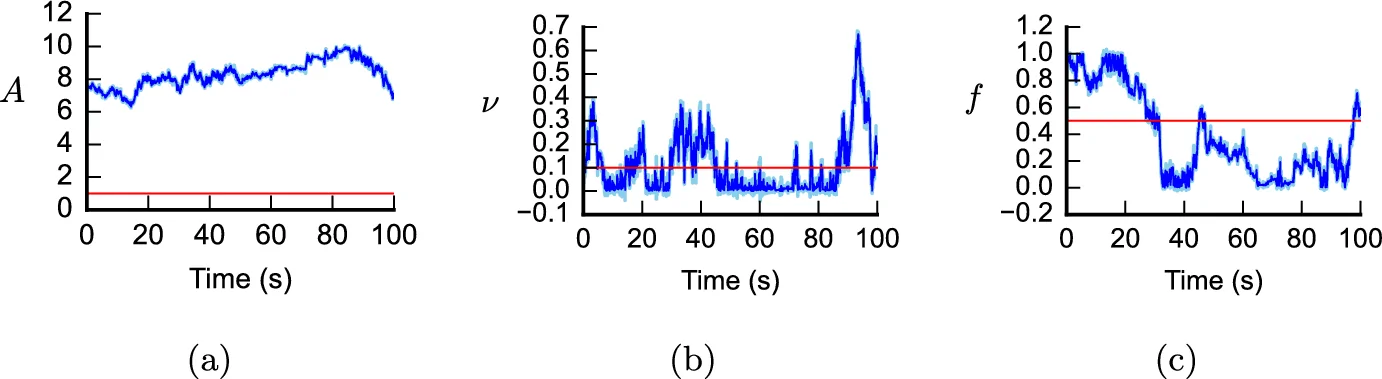
q=1,R=0.5

**Fig. 21:**
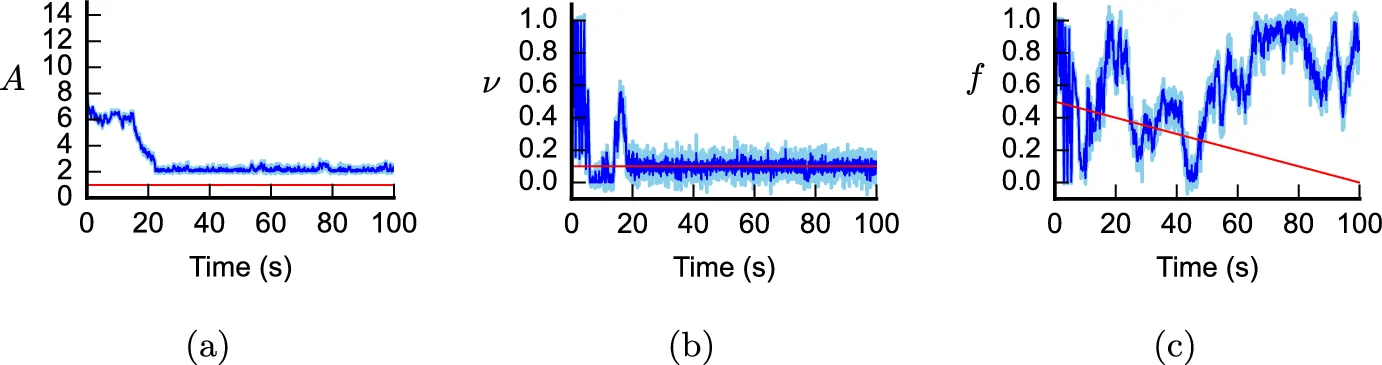
For the same numerical experiment as Fig. 1 except for changing the prior of *A* to $\mathcal {U}([0,2])$ which does not include the true value $A^*=1$, we show the sequence of empirical posterior means (blue line) within 2σ band (shaded region) compared to their true values (red line) (color figure online)

**Fig. 22:**
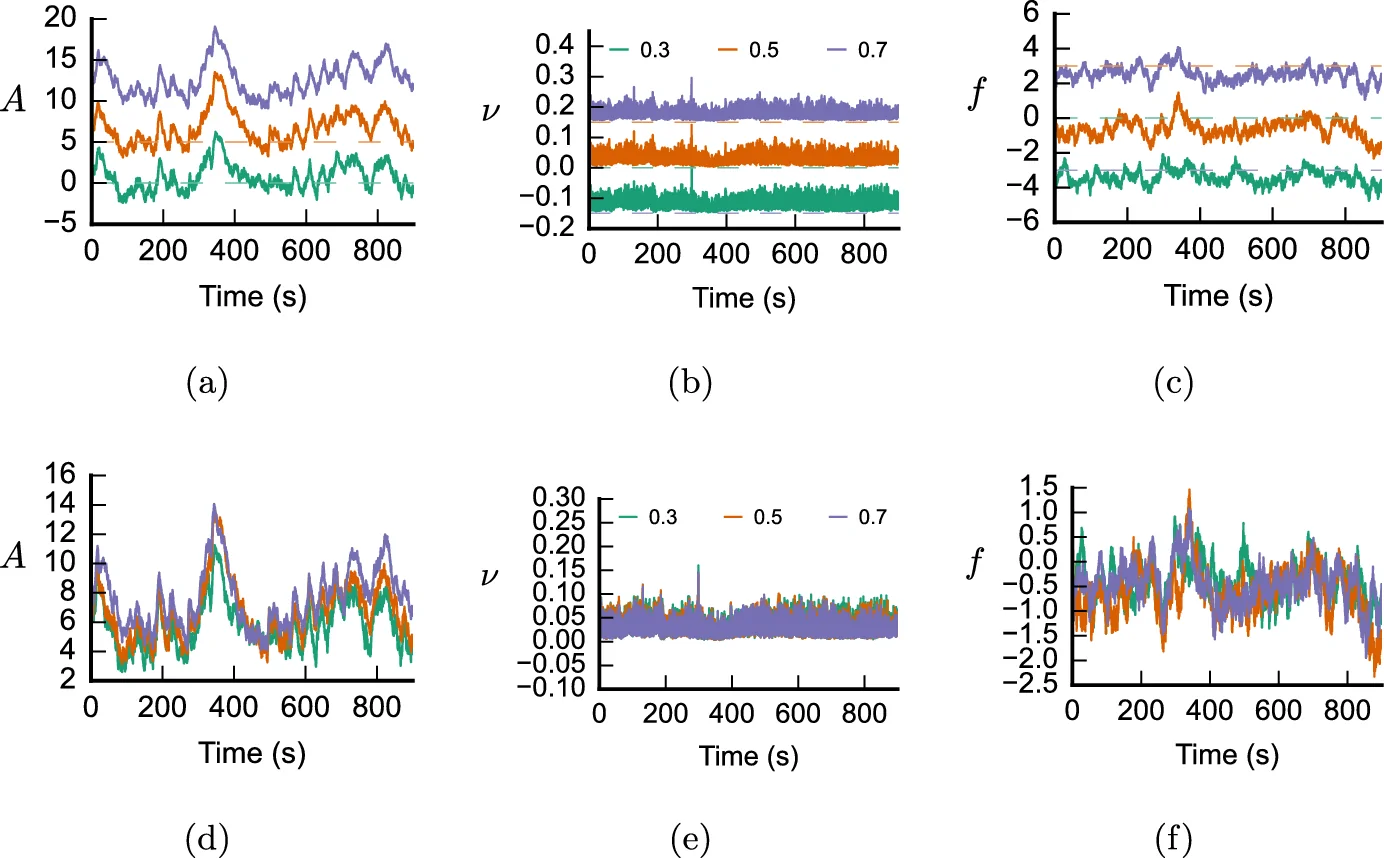
Inferred parameters for $\alpha =60^\circ$ for three tapering fractions of the Tukey window. The top row shows the trajectories with an offset

## Data Availability

Imaging data used in this work have been deposited in the Donders Repository https://doi.org/10.34973/2w2s-tg07.

## Appendix A Sensitivity analysis

We perform a preliminary sensitivity analysis to assess the influence of the input parameters θ on the neural field dynamics. We proceed in three stages. First stage: we simulate the spatio-temporal field *u*(*x*, *t*) by numerically integrating the semi-discrete system Eq. (4) across an ensemble of $N_s=2560$ parameter combinations θ. This ensemble is sampled from the parameter range $\theta =(A,\nu ,f)\in [0,10]\times [0,1]\times [0,1]$ using Saltelli’s extension of the Sobol sequence; this same range will be used for the priors when inferring these parameters. All simulations of Eq. (4) are on a spatio-temporal grid [0,10]×[0,100] with dr=0.02 and dt=0.005, with τ=1, a=0 and the rest of the model parameters are as in Table 1. Second stage: each realization of these two-dimensional fields is reduced into a scalar quantity of interest (QOI) via Singular Value Decomposition; we decompose each field into modes using

$$\begin{aligned} U=\Phi \Sigma \Psi , \end{aligned}$$

where $\Phi =(\Phi _1, \cdots ,\Phi _J,)$ are the spatial modes, $\Psi =(\Psi _1, \cdots ,\Psi _J)$ are the temporal modes and $\Sigma =\text {diag}(\sigma _1,\cdots ,\sigma _J)$ are the singular values with $\sigma _1\ge \sigma _2\ge \cdots$, we then extract the following scalar quantities of interest:

1. $\eta _1$ fraction of energy of the first mode, given by $\eta _1=\displaystyle \frac{\sigma _1^2}{\sum _{i=1}^{J}\sigma _i^2}$;
2. $k_{90}$ number of modes accounting for 90% of energy, calculated as
  $$ k_{90}=\min \Biggl \{k: \displaystyle \frac{\sum _{i=1}^{k}\sigma _i^2}{\sum _{i=1}^{J}\sigma _i^2}>0.9\Biggr \}; $$
3. $R_1$ roughness of the first spatial mode, given by $R_1=\Vert \nabla \Phi _1\Vert ^2_2$;
4. $T_1$ period of the first temporal mode, given by the inverse of the dominant frequency $T_1=1/\arg \max _f \vert \hat{\Psi }_1\vert ^2$.

We use these different quantities of interest (rather than a single one) to examine different aspects of the model dynamics (energy, frequencies, ...), which yields a global picture of the model’s sensitivity structure.

Third stage, we compute the first order *S*1 and total order *ST* Sobol indices of the parameters *A*, ν and *f*. *S*1 and *ST* measure the individual and total contribution of each input (parameter) to the output (QOI) variance respectively. We report their values in Table 3.

**Table 3:** First order (S1) and total order (ST) Sobol indices for parameters *A*, ν and *f* drawn from $[0,10]\times [0,1]\times [0,1]$, and for scalar quantities of interest $\eta _1$, $k_{90}$, $R_1$ and $T_1$

	$\eta _1$		$k_{90}$		$R_1$		$T_1$	
	ST	S1	ST	S1	ST	S1	ST	S1
*A*	0.784	0.405	0.805	0.1	0.368	0.076	0.748	0.355
ν	0.161	0.063	0.303	0.063	0.903	0.493	0.298	0.082
*f*	0.443	0.065	0.823	0.061	0.282	0.028	0.479	0.127

The three parameters exhibit moderate to high total indices *ST* and gap ST-S1 across all QOIs, which indicates that each parameter is influential mainly through its interactions with other parameters and not on its own. *A* has large *ST* and moderate *S*1 for all QOIs except $R_1$, which indicates that this parameter is very influential, mainly through its interactions with the other parameters. ν primarily controls $R_1$ both directly and through interactions, and influences the other QOIs mainly through interactions as its *S*1 is negligible. *f* appears to have almost no independent effect as its *S*1 is negligible compared to *ST* for all QOIs. For the parameter inference task, this suggests that the observations can constrain combinations of the parameters, not each separately. However considering a smaller range for parameters, around the true values from the experiments of Sect. 3 ($A=1,\; \nu =0.1, f\in [0,0.5]$), paints a different picture for the parameter *f* in particular. We calculate total and first order Sobol indices for parameters in the range $\theta =(A,\nu ,f)\in [0.5,1.5]\times [0.05,0.5]\times [0,1]$, while keeping the same setting as before. The values are reported in Table 4.

**Table 4:** First order (S1) and total order (ST) Sobol indices for parameters *A*, ν and *f* drawn from $[0.5,1.5]\times [0.05,0.5]\times [0,1]$, and for scalar quantities of interest $\eta _1$, $k_{90}$, $R_1$ and $T_1$

	$\eta _1$		$k_{90}$		$R_1$		$T_1$	
	ST	S1	ST	S1	ST	S1	ST	S1
*A*	0.182	0.066	0.309	0.018	0.344	0.018	0.358	0.060
ν	0.044	0.0145	0.162	0.027	0.574	0.036	0.712	0.365
*f*	0.936	0.824	1.038	0.595	0.997	0.239	0.628	0.260

In this restricted range, the three parameters exhibit different behaviours: *A* has negligible first order indices *S*1 across all quantities of interest, but moderate total indices *ST*. ν has negligible *S*1 and *ST* except for $R_1$ where it influences mainly through interactions, and $T_1$ for which it has high *S*1 and *ST*; finally, *f* seems to be dominant for all four quantities of interest through its interactions as well as directly. This exploration of the sensitivity near the true values of the parameters suggests that once the particles approach this restricted region in the parameters space, inference of the parameters should be easier, since the influence of the parameters is more direct.

## Appendix B Robustness

### B.1 Discretization parameters

While an extensive study of the effect of discretization schemes on inference is beyond the scope of this paper, we present some evidence that our results are robust to changes in the temporal and spatial discretization parameters Δt=0.01 and J=30 used in Sect. 3. We only require that these steps permit us to resolve the frequencies present in the observations. For this purpose, we conduct the same experiment as described in Sect. 3 while only changing Δt and *J*. In Fig. 14, we show the effect of using a Δt that is double the one used in Sect. 3. We observe that the posterior mean estimates of *A* and ν still converge to the true value, while *f* exhibits a similar behaviour to Fig. 1c for the first $60\textrm{s}$, after which its deviations from the truth are higher than in Fig. 1c. In Fig. 15, where Δt is half the one used in Sect. 3, the estimates of *A* and ν behave the same as in Fig. 1a and b. However, the estimate of *f* for this smaller Δt seems to track more closely the temporal frequency variations exhibited by the state in Fig. 2c. Figure 16, which corresponds to a smaller state dimension *J* (a bigger spatial discretisation step Δr), shows slower convergence for *A* and ν with more fluctuations around their true values, while the estimate for *f* manages to remain relatively close to the truth until around $60\textrm{s}$ after which it diverges. In Fig. 17, increasing *J* (smaller Δr) yields posterior mean estimates of *A* and ν that converge almost instantly with small and stable variance, and a behaviour similar to Fig. 1c for *f*. Across these tests, the posterior mean estimates of *A* and ν remain stable across varying and reasonable discretisation resolutions. As for *f*, the discretisation steps must be sufficiently small to ensure that the numerical integration of the evolution model captures the observed dynamics of this parameter.

### B.2 Noise intensities

The performance of particle filters is highly sensitive to process and observation noise intensities, though the two play different roles. In fact, *Q* governs the spread of the ensemble of particles in the prediction step. If *Q* is too small, the particles remain tightly clustered and fail to cover the region of the space where the true state is, which leads to an impoverished sample after resampling. If *Q* is too large, the ensemble will be overly spread and therefore the filter estimates lose precision. *R* controls the sharpness of the likelihood and therefore the weighting of the particles. If *R* is too small, non-negligible weights will be assigned to a handful of particles which will then be duplicated while discarding the rest of the ensemble through resampling, leading to a loss of diversity. On the other hand, a very large *R* will result in all particles receiving similar weights regardless of their proximity to the observation, making the later non informative. This misspecification of the noise intensities for the inner layer of the nested particle filter can directly distort the outer weights of the parameters, since these are calculated from the state weights (Algorithm 2, line 13). Practically, this can manifest in pathological behaviours such as weight collapse or an invariant particle ensemble that does not update with new observations. To illustrate this, we run the same experiment as Sect. 3 for different *Q* and *R* pairs while eliminating the model mismatch aspect. That is, we set $a^*$ to 0 for the process generating observations. Figures 18, 19 and 20 show some examples of the effect of misspecified *Q* and *R* on the parameter inference. For Fig. 18, after an initial period where the estimates are near the true values, the filter diverges and the variance blows up. In Fig. 19, the ensemble is not moving and particle weights are not updated after each new observation. As a result, the estimates are stuck at the mean of the prior with a variance that does not shrink as new information accumulates. As for Fig. 20, the filter settles near the wrong values with almost zero variance, indicating a possible ensemble collapse to few particles.

### B.3 Prior range

The choice of the prior range for the parameters and the jittering kernel is also critical. A prior that does not include the true value, or has insufficient initial coverage near it will result in a failed recovery of the parameters. By (14), the parameter particles are constrained to vary in the range dictated by the prior ( which is also the support of the jittering kernel), they cannot escape this range regardless of what the observations may suggest. If the true value is not included in the initial range, the filter will not recover it and will produce an incorrect estimate. This failure can manifest itself as the particles, and by consequence the posterior mean, clustering near the boundary of the prior range closest to the true value. This can have further consequences for the estimation of the parameters: the filter may partially compensate for a misplaced parameter by adjusting the estimation of one or more other parameters in an effort to make the predicted state close to the observations. To illustrate this, we repeat the same experiment as Sect. 3 while changing only the prior range of *A* to from $\mathcal {U}([0,10])$ to $\mathcal {U}([2,10])$ which excludes the true value $A^*=1$. Figure 21 shows that while we successfully recover ν, the posterior mean of *A* stalls near the boundary of its prior while *f* varies to absorb the mismatch between the state and observations due to the error in *A*. A plausible choice of the prior should then be guided by physical knowledge of the parameter (known bounds, typical magnitude, etc.) and the characteristics of the observations and how they are related to the parameters.

**Table 5:** RMSE and $\Delta f_t$ for different tapering fractions β of the Tukey window for $\alpha =60^\circ$

	RMSE	$\Delta f_t$
β=0.3	2.2565	0.0150
β=0.5	2.0712	0.0156
β=0.7	2.0554	0.0141

### B.4 Tukey window

The tapering fraction β of the Tukey window used as observation operator in the experiments of Sect. 5, controls how much of the signal is preserved without modification and how much is subject to decay at the edges. In order to evaluate the effect of β on the dynamics of the estimated parameters and quality of state estimation, we run experiments in the same setting described in Sect. 5 for angle $\alpha =60^\circ$ for different values of the tapering fraction β=0.3,0.5,0.7. Figure 22a–c show the posterior means of the inferred input parameters with added/ subtracted offset to separate them, while Fig. 22d–f show them overlaid. These figures show that the dynamics of the mean posteriors are globally conserved through the change of β, with the amplitude parameter *A* slightly decreasing with β and *f* showing some slight variations between the different values of β. We report the aggregated ${{\,\textrm{RMSE}\,}}$ and frequency deviation $\Delta f_t$ for the three cases in Table 5. While $\Delta f_t$ remains almost unchanged, the ${{\,\textrm{RMSE}\,}}$ for β=0.5 and 0.7 is lower which indicates these values are more suitable for our application.

## Code availability

A repository with codes is available at https://doi.org/10.5281/zenodo.21795156.
